# Porous Alumina Films Fabricated by Reduced Temperature Sulfuric Acid Anodizing: Morphology, Composition and Volumetric Growth

**DOI:** 10.3390/ma14040767

**Published:** 2021-02-06

**Authors:** Alexander Poznyak, Andrei Pligovka, Tsimafei Laryn, Marco Salerno

**Affiliations:** 1 Department of Electronic Technology and Engineering, Belarusian State University of Informatics and Radioelectronics, 6 Brovki Str., 220013 Minsk, Belarus; poznyak@bsuir.by; 2 Research and Development Laboratory 4.10 “Nanotechnologies”, Belarusian State University of Informatics and Radioelectronics, 6 Brovki Str., 220013 Minsk, Belarus; tilar2001@tut.by; 3 Department of Micro- and Nanoelectronics, Belarusian State University of Informatics and Radioelectronics, 6 Brovki Str., 220013 Minsk, Belarus; 4 Department of Functional Materials and Hydrogen Technology, Military University of Technology, 2 Kaliskiego Str., 00-908 Warsaw, Poland; marco.salerno@wat.edu.pl

**Keywords:** sulphuric acid, galvanostatic anodizing, dissociation, X-ray diffraction, current efficiency, anodic aluminum oxide (AAO), aluminum foil, Pilling–Bedworth ratio, low current density

## Abstract

The volumetric growth, composition, and morphology of porous alumina films fabricated by reduced temperature 280 K galvanostatic anodizing of aluminum foil in 0.4, 1.0, and 2.0 M aqueous sulfuric acid with 0.5–10 mA·cm^−2^ current densities were investigated. It appeared that an increase in the solution concentration from 0.4 to 2 M has no significant effect on the anodizing rate, but leads to an increase in the porous alumina film growth. The volumetric growth coefficient increases from 1.26 to 1.67 with increasing current density from 0.5 to 10 mA·cm^−2^ and decreases with increasing solution concentration from 0.4 to 2.0 M. In addition, in the anodized samples, metallic aluminum phases are identified, and a tendency towards a decrease in the aluminum content with an increase in solution concentration is observed. Anodizing at 0.5 mA·cm^−2^ in 2.0 M sulfuric acid leads to formation of a non-typical nanostructured porous alumina film, consisting of ordered hemispheres containing radially diverging pores.

## 1. Introduction

Electrochemical anodizing in various aqueous acid solutions results in porous alumina films (PAFs) having a quasi-regular nanoscale cellular structure of pores, which occurs naturally as a result of self-organization [1,2]. The spontaneously emerging structure can be improved with the help of more [3] or less [4,5] complex techniques, which also make it possible to form PAFs with unusual morphology [6]. Thanks to its “technological plasticity” and the relative simplicity of formation, porous alumina is a promising material for a wide range of applications, such as an electronic product elemental base [7,8,9,10,11], or in various types of sensors and detectors [12,13,14,15,16]. A currently rapidly developing field is the creation of nanoscale structures and composite materials using porous anodic aluminum oxide as a highly ordered nanostructured matrix for the formation of various kinds of functional materials and devices [17,18,19,20,21,22,23,24,25,26], including nanostructures such as carbon nanotubes, nanodiamonds, and graphene [27,28,29]. The above-described PAFs implementations require research results on the effect of the electrolyte nature, electrical anodizing modes, and other factors on the cellular–porous structure parameters and the PAF properties both in traditional electrolytes [30,31,32,33,34,35,36,37,38,39,40] in various compositions [41,42,43] and unconventional acidic solutions [44,45,46,47,48,49,50,51]. It is known, for example, that galvanostatic anodizing of aluminum in acidic electrolyte can occur at different rates, depending on the electrolyte nature and concentration, and on the anodic current density [30,38]. Upon anodizing completion, the formed PAF thickness exceeds the thickness of the pristine aluminum layer. This phenomenon is called volumetric growth [31,32,37] and is quantitatively described by one quantity named with various synonyms: “Pilling–Bedworth ratio”, “volume expansion factor” [34,40,41,52,53], “volumetric growth factor” [45,46], or “thickness expansion factor” [35]. However, the numerical values experimentally found in the literature for this parameter are sometimes inconsistent, and the respective experiments are not always comparable with each other [31,32,35,37,41,51,52,53,54,55]. At the same time, reliable knowledge about the parameters of the PAF cellular structure, thickness, and properties is required to create various types of materials and devices. In this regard, over the years Dr. Viktor Surganov and coworkers have undertaken systematic comprehensive investigation of the PAF formation, dissolution, and growth in solutions of oxalic [54,55], orthophosphoric [56,57], malonic [44,46], and sulfosalicylic [45] acids, under comparable conditions. The experimental techniques and the aluminum quality in the other author investigations for sulfuric acid (SA) anodizing of aluminum [35,40,41,58] are quite different, which makes it difficult to compare the results. For example, in ref. [40], aluminum foil of 98.0% purity was used, and no detail was given about the nature of the impurities and the samples pre-treatment (for example, whether annealing was carried out in order to homogenize the alloy). In ref. [41], 1050 A aluminum alloy (99.5% purity) was used, whereas in ref. [35], aluminum foil of 99.9% purity as well and aluminum films deposited on silicon wafers (without detailed characterization) were used as initial samples.

A very comprehensive investigation of PAF fabrication in SA was carried out in ref. [34], which presented outstanding PAF volume growth and richness of experimental methods. The galvanostatic anodized aluminum was designed in model compositions consisting of sequentially sputter-coated aluminum and aluminum–tungsten alloy, aluminum foil, and sputter-coated aluminum, at SA concentrations of 0.4 and 2.55 M and temperatures of 0 and 20 °C, in the current density range of 0.5–50 mA·cm^−2^. One of the goals of the present work was to carry out similar anodizing on single film aluminum and check if similar results as obtained in that work also occur in this case.

This work is devoted in particular to the experimental determination of the PAF volumetric growth rate and efficiency for low temperature aluminum anodizing in aqueous SA electrolyte at different concentrations of 0.4, 1.0, and 2.0 M and current densities in the range of 0.5–10 mA·cm^−2^. PAF scanning electron microscopy (SEM) and X-ray diffraction (XRD) were performed, the results of which made it possible to estimate the amount of metallic aluminum after the end of the anodizing.

One characteristic feature of this study is the expression of the electrolyte concentration not in mass fractions, as for example in [40], but in mol·dm^−3^, which in the authors’ opinion is more correct, since it actually characterizes the chemical amount of equivalent substances present in the electrolyte. Secondly, the investigation was carried out in a pure SA solution containing no additives, as in refs. [41,43]. Thirdly, the study is methodologically comparable with the previously conducted studies for solutions of oxalic and orthophosphoric acids [54,55,56,57]. Additionally, the results are absolutely comparable with those obtained earlier for malonic and sulfosalicylic acid solutions [44,45,46], since they were obtained under similar conditions using aluminum specimens from the same set of samples. The combined assessment of the anodizing rate, current efficiency of anodizing, and residual amount of un-oxidized aluminum distinguishes the present study from similar researches carried out in recent years [34,35,40,41].

## 2. Materials and Methods

Experimental samples were prepared from aluminum foil (99.99% purity) with 10.5 µm thickness. The sample contact area was masked with barrier anodic oxide formed in a 1% wt. aqueous citric acid solution by the potentiodynamic anodizing with voltage sweep speed of 2 V·s^−1^ to 290 V, followed by exposure at 290 V in potentiostatic mode. The quality of the isolating film was considered sufficient when the current density decreased below 1% of its initial value. A part of the sample protected by barrier oxide served to provide electrical contact with power source and prevent a meniscus effect; the rest was immersed in a solution of sulfuric acid and was then completely anodized. The anodized area was 4 cm^2^; the process was carried out in a galvanostatic mode, varying the current density in the range of 0.5–10 mA· cm^−2^. Anodizing was carried out in a glass electrochemical cell with an aluminum cathode. As for the electrolytes, we used 600 mL SA aqueous solutions with 0.4, 1.0, and 2.0 M concentration. The temperature was maintained with the highest possible accuracy at 280 K using an ice bath, the spread not exceeding ±1 K. SA was supplied by the Belaquilion additional-liability company and manufactured by Sigma-Aldrich, Inc. A programmable power supply 5751 A (Keysight Technologies Inc., Santa Rosa, CA, USA) was used as the anodizing unit, controlled by a personal computer (PC) with homemade software written in LabVIEW 2018 (National Instruments Corp., Austin, TX, USA). Programmable digital multimeters 34470 A (Keysight Technologies Inc., Santa Rosa, CA, USA) were used to record the voltage–time responses, controlled by a PC with homemade software written in LabVIEW 2018.

The process was stopped when the anodizing voltage *E_a_* rose steeply, meaning that the aluminum foil was oxidized completely. The anodizing time *τ* of the samples until complete anodizing was measured, and the aluminum anodizing rate was calculated as:

(1) $$V_{a}=\frac{h_{Al}}{\tau }$$

where *h_Al_* is starting aluminum foil thickness.

To determine both the starting aluminum foil and the resulting PAF thickness, a digital micrometer Micromar 40 EWR (Mahr Inc., Providence, RI, USA) was used. The PAFs were observed in a SEM S-4800 (Hitachi High-Technologies Corporation, Tokyo, Japan) operated at 10–15 kV, after over coating the specimens with a thermally evaporated 3 nm thick gold layer to reduce the charging effects.

The volumetric growth factor *K_g_* was calculated [24,41,44] as:

(2) $$K_{g}=\frac{h_{PAF}}{h_{Al}}$$

The current efficiency *η* [44] was determined from the theoretical charge *Q_ox_*(*th*) required to oxidize the whole aluminum foil, according to the reaction Al—3e^−^→Al^3+^ and from the electric charge:

(3) $$Q_{ox}(real)=j_{a}\cdot \tau$$

where *τ* is the corresponding time for complete anodizing and *j_a_* is the anodizing current density, as obtained from the experimental voltage-time responses. After above definitions, the following relation applies, for the anodizing efficiency (%):

(4) $$\eta =\frac{Q_{ox}(th)}{Q_{ox}(real)}$$

Investigations of the PAFs composition were carried out by XRD performed on a diffractometer DRON-3 (Bourevestnik, JSC, St. Petersburg, Russia) connected to PC, Cu-Kα-radiation with graphite filter.

For convenient handling, the brittle PAF samples were glued to a glass substrate with BF-2 glue, and then were fixed in a holder.

Graphical dependencies and curve-fittings were developed using OriginPro 2018 by OriginLab Corporation (Northampton, MA, USA).

## 3. Results and Discussion

**Figure 1:**
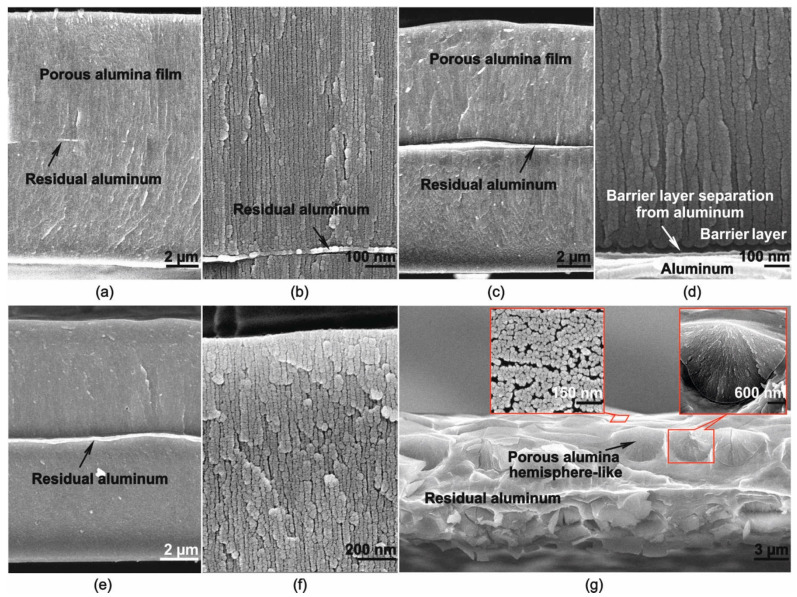
(**a**,**b**) SEM images of porous alumina films fabricated by low temperature sulfuric acid anodizing at current densities of (**a**,**b**) 10, (**c**,**d**) 4.9, (**e**,**f**) 0.9, (**g**) 0.5 mA·cm^−2^ and concentrations of (**a**,**b**,**g**) 2.0 and (**c**–**f**) 0.4 M.

Figure 1 shows cross-section SEM images of the most relevant and representative PAF samples. Clearly, all the shown samples except that one formed at 0.5 mA·cm^−2^ have a cellular-porous structure with pores oriented normally to the surface. The microscope resolution was insufficient to accurately assess the pore diameter and the periodicity of the cellular structure.

All SEM images also present minor amount of residual aluminum. This is probably due to the fact that the aluminum anodizing was uneven over the macroscopic foil surface, and there was some other path allowed for the current to pass, elsewhere than the currently shown sample area. In Figure 1d, one can also see that detachment of the barrier layer occasionally occurred as a result of PAF preparation for SEM.

**Figure 2:**
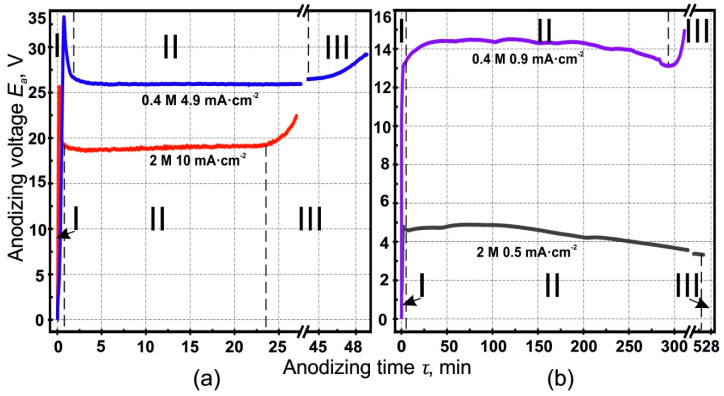
Voltage-time responses of low temperature anodizing aluminum foil at current densities of (**a**) 10, 4.9, and (**b**) 0.9, 0.5 mA·cm^−2^, and sulfuric acid concentrations of 0.4 and 2.0 M. The profiles have been presented in two separate panels, for the sake of clarity.

Figure 2a,b shows the chrono-potentiometric profiles of some representative anodizing.

All curves have a typical form where three consecutive time regions (I-III) can be distinguished, with a pronounced intermediate stationary regime, where the profile is roughly horizontal (i.e., parallel to the time axis), apart from minor deviations possibly due to fluctuations in either temperature or local ionic concentration. The first region I, in which the anodizing voltage rises to its maximum and the main reason for the voltage increase at stage I is the growth of the barrier layer thickness. Then one can observe the voltage to stay smoothly at a stationary level, which corresponds to the pore formation process [37]. In this regime, nucleation of the pores occurs at the sites of defects and at the grain boundaries. Usually, many pore embryos form that compete for a position, until some embryos absorb other surrounding ones, forming a pore [34,59].

At the end of region I, a full-fledged cellular structure is formed with well-defined pores diameter and periodicity. The second region II, stationary, characterizes the growth of pores in depth, i.e., the increase in PAF thickness. The voltage *E_a_* of the stationary region II gives information about the periodicity (cell size or interpore distance) *D_int_* and the pore diameter *D_por_* [30,50,60].

In region III, the voltage begins to increase smoothly when the aluminum starts to be locally consumed and transverse anodizing occurs. At the moment when most aluminum has been consumed, maintaining the current at a stationary level becomes impossible, since the PAF is insulating, and as a result the voltage increases. Region III was not found for the anodizing parameters combination of 0.5 mA·cm^−2^ and 2.0 M. This is probably due to non-typical processes occurring under these anodizing conditions, resulting in the non-typical PAF structure shown in Figure 1g. In this case, pits are visible in the form of hemispheres, with internal substructure of co-existing pores. Analyzing the shape of one such hemisphere, one can assume that its nucleation and development began from one central point. Probably, at low values of the current density, when the dissolution of aluminum is significant [34], the continuous film formation is difficult and the current density is insufficient for nucleation at the initial stage of the cellular–porous structure on the entire surface. We assume that under these conditions a self-organized redistribution of the current density over the sample surface occurs, in such a way that pores nucleation does not occur over the entire surface but only in some places, which is not typical for classical PAF. It is not yet clear to what extent such nucleation is ordered or chaotic, and this requires additional research. However, apparently a high degree of internal ordering occurs.

**Figure 3:**
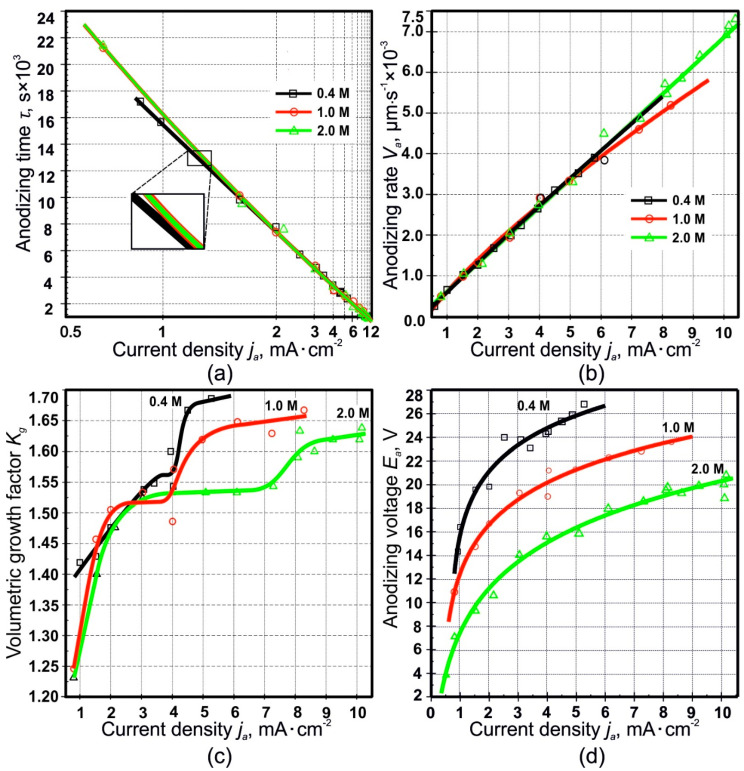
Dependence of the time *τ* of full anodizing (**a**), anodizing rate *V_a_* (**b**), volumetric growth factor *K_g_* (**c**), and anodic voltage of stationary region II *E_a_* (**d**) on current density *j_a_* of porous alumina films samples fabricated at current densities *j_a_* of 0.75–10 mA·cm^−2^ and sulfuric acid concentrations of 0.4–2.0 M.

The experimental dependencies of *τ*,$V_{a},K_{g},E_{a}=f\left(j_{a}\right)$ are shown in Figure 3.

The experimental trends observed in Figure 3a,b and d have been modeled, and the respective datapoints have been fitted, with empirical formulas, chosen as follows:

(5) $$\tau =\frac{a}{1-b\cdot j_{a}}$$

(6) $$V_{a}=A+B\cdot j_{a}{}^{C}$$

(7) $$E_{a}=\alpha +\beta \cdot \ln\left(\frac{j_{a}+\gamma }{\delta }\right)$$

where *a*, *b*; *A, B*, *C*; *α, β*, and *γ* are empirical constants depending on the SA concentration. The coefficient *δ* in front of the argument of the natural logarithm is required to comply with both requirements: having the actual argument to be a pure number; and having the *j_a_*—and the homogeneous quantity *γ* summed with that—expressed in actual physical units. The best fitting values for these empirical constants are presented in Table 1.

Stationary PAF growth is found at current densities not exceeding 5.5, 8.5, and 10.1 mA·cm^–2^ in 0.4, 1.0, and 2.0 M SA, respectively. The existence of these threshold current densities is due to microscale dielectric breakdowns occurring in the PAF. For solutions of sulfuric acid and mixtures of solutions of sulfuric acid and sulfates, a combination of these parameters can be found in [61]. This phenomenon is also known for other electrolytes [44,46,57]. This can be explained as follows. The increase in current density leads to a decrease in the thickness of the barrier oxide. The cause is attributed to a higher electric field, which acts on the barrier oxide and thus leads to an increase in the field-induced oxide dissolution. This is supported by a thermally induced oxide dissolution, but the higher anodizing current induces Joule heating and a larger local temperature increase at the bottom of the pores. This is explained by a change in the distribution of electric field on the pore bottoms, caused by a stronger field-induced dissolution of the barrier oxide in a stronger or more concentrated acid [61,62,63]. Therefore, when the thickness of the barrier layer reaches a critical value, a microscale dielectric breakdown occurs.

**Figure 4:**
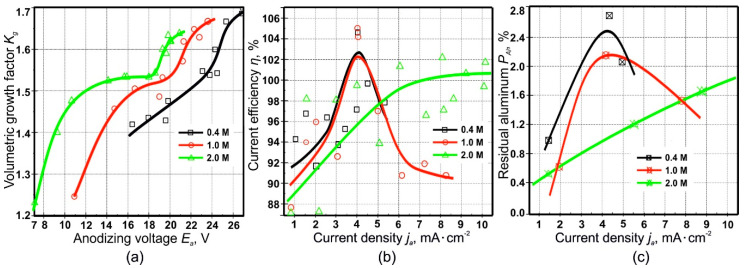
(**a**) Dependence of volumetric growth factor *K_g_* on anodic voltage *E_a_* in stationary growth region II of porous alumina films samples fabricated at current densities *j_a_* of 0.5–10 mA·cm^−2^ and sulfuric acid concentrations of 0.4–2.0 M. Dependence of current efficiency *η* (**b**) and fraction of un-oxidized aluminum *P_Al_* (**c**) on the anodic current density for sulfuric acid concentrations of 0.4, 1.0, and 2.0 M. The lines are just guides to the eye.

Figure 3b shows that the anodizing rate increases with increasing current density, and the dependence can also be described by another empirical formula, i.e., Equation (6), with three fitting parameters. An increase in the anodizing rate with an increase in the anodic current density is obvious; theoretically, the dependence should be linear, but surprisingly there is a noticeable deviation from linearity for the case of 1.0 M SA. This effect can be explained by two reasons. Firstly, with an increase in the anodic current density, an increasing deviation of the anodizing from equilibrium is likely to occur, and the charge is increasingly consumed in side processes, which in turn leads to an increase in the anodizing time and a decrease in the anodizing rate in comparison with the theoretical one. Secondly, when taking into account the decrease in proportion of un-oxidized aluminum (Figure 4c), an increase in current density may result in additional charge (and, accordingly, time) being spent on the anodizing.

In Figure 3c, one can see that an increase in current density and a decrease in SA concentration both contribute to an increase in PAF volumetric growth. It should be noticed that the form of these dependences differs from those described in refs. [44,46,54,55,56], which should likely be similar, despite corresponding to anodizing carried out in different electrolytes. The main difference lies in the presence of two saturation regions in the curve, instead of one [45,54,55,56] or three [44,46]. The beginning of the first saturation region at any SA concentration corresponds to a current density of about 2.0–2.5 mA·cm^−2^, while at the same time, the extension of the first saturation region along the abscissa grows with an increase in SA concentration. The second saturation region begins at current densities above 4.5, 5, and 8 mA·cm^−2^ for 0.4, 1.0, and 2.0 M concentrations, respectively, which is also clearly visible in Figure 3c. Such a *K_g_* stepwise dependence for SA was observed formerly for the case of aluminum anodizing in malonic acid [44,46]. At the same time, the tendencies of the *K_g_* dependence on the anodizing conditions coincide with those described in refs. [34,40], and the values are generally close, although an accurate estimate is difficult due to the difference in the experimental conditions.

The change in PAF volumetric growth can be influenced mainly by two factors. Firstly, the negatively charged anions and anionic complex compounds from the electrolyte are adsorbed by the oxide surface and are partially incorporated into the PAF, which leads to its “swelling”, as unambiguously confirmed by the results on incorporation of sulfur species from SA presented in ref. [35]. Secondly, the electrolyte and the PAF outer layer interact chemically, which leads to partial etching of the PAF. At low current densities in aggressive electrolytes such as SA [64], the aluminum oxidation rate is low, the contact time with electrolytes is long, and the dissolution rate is comparable to the oxidation rate. A sharp decrease of *K_g_* at long contact times (low anodic current densities) as observed here, also according to the data of ref. [34], confirms this line of reasoning. Something similar was noted in ref. [65] during anodizing in a fluoride-containing electrolyte, which exhibited strong aggressiveness towards the formed oxide, such that in certain cases PAF turned out to be almost completely etched away, i.e., its thickness decreased significantly.

The authors did not study the dependence of the volumetric growth on temperature, but suggest that it should also exist. The ref. [61] also shows that an increase in the anodizing temperature leads to a decrease in the anodizing voltage and a noticeable increase in the pore diameter and to reduce the thickness of the anodic oxide layer. Thus, anodizing at higher temperatures should lead to a decrease in volume growth. This assumption is all the more valid, since the ref. [66] thoroughly demonstrates the influence of the electrolyte type and temperatures on the anodizing process characteristics and morphological parameters of the formed oxide.

**Figure 5:**
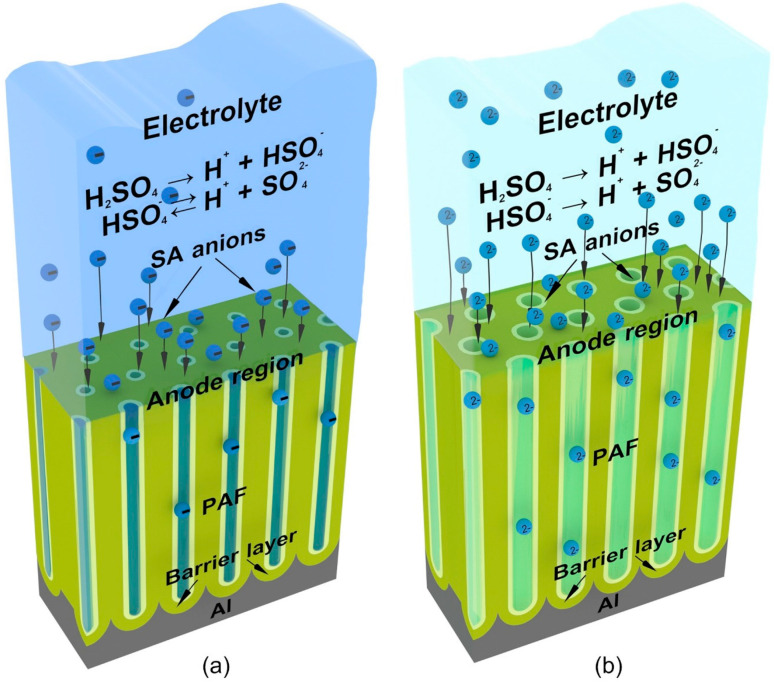
Schematic 3-D views of the formation process of porous alumina films in sulfuric acid solutions with concentration increased (**a**) and reduced (**b**) with respect to the starting value.

The presence of two saturation regions (Figure 5) can be explained by the fact that SA is strong and dibasic, and at the first stage, it dissociates almost completely, while the second proton elimination is facilitated in more dilute solutions at lower values *j_a_* or in more concentrated solutions with a significant increase in current density. Since the dissociation in the second stage is suppressed with increasing concentration, the amount of acid residue anions incorporated into the PAF from concentrated solutions may be even less than in dilute solutions. A current density increase, leading to the growth of *E_a_* as shown in Figure 3d (the dependence of which on *j_a_* is represented by Equation (7)), and thus the increase in anions concentration close to the anode, also contributes to an increase in number of Al^3+^ ions ejected into the solution. The result may be an increase in aluminum anionic complex compounds concentration in the anode region. The possibility of the field-assisted ejection of Al^3+^ ions, with no requirement for field-assisted dissolution of the PAF, is also pointed out in refs. [31,34,67,68]. As seen in Figure 3d, an increase in electrolyte concentration results in a decrease in anodic voltage. This can be explained by two reasons. Firstly, the electrical conductivity of the more concentrated SA solutions may be higher, and secondly, the more concentrated solutions cause a lower thickness and therefore less resistance of the barrier oxide.

Figure 4a shows the volumetric growth factor dependence on the anodic voltage, which has similar trend as the dependence $K_{g}=f\left(j_{a}\right)$ presented in Figure 3c. A similar dependence of volumetric growth factor *K_g_* on anodizing voltage *E_a_* in several other conditions was found in ref. [58].

**Figure 6:**
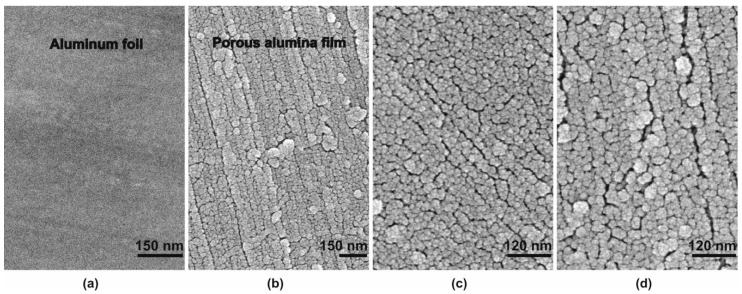
SEM images of the surface of (**a**) initial aluminum foil and porous alumina films fabricated by low temperature sulfuric acid anodizing at current densities *j_a_* of (**b**) 10, (**c**) 4.9, and (**d**) 0.9 mA·cm^−2^ and sulfuric acid concentrations of (**b**) 2.0 and (**c**,**d**) 0.4 M.

A decrease of *K_g_* in concentrated solutions due to chemical dissolution of the PAF surface at low temperature is unlikely, which is confirmed by the SEM images presented in Figure 6.

**Figure 7:**
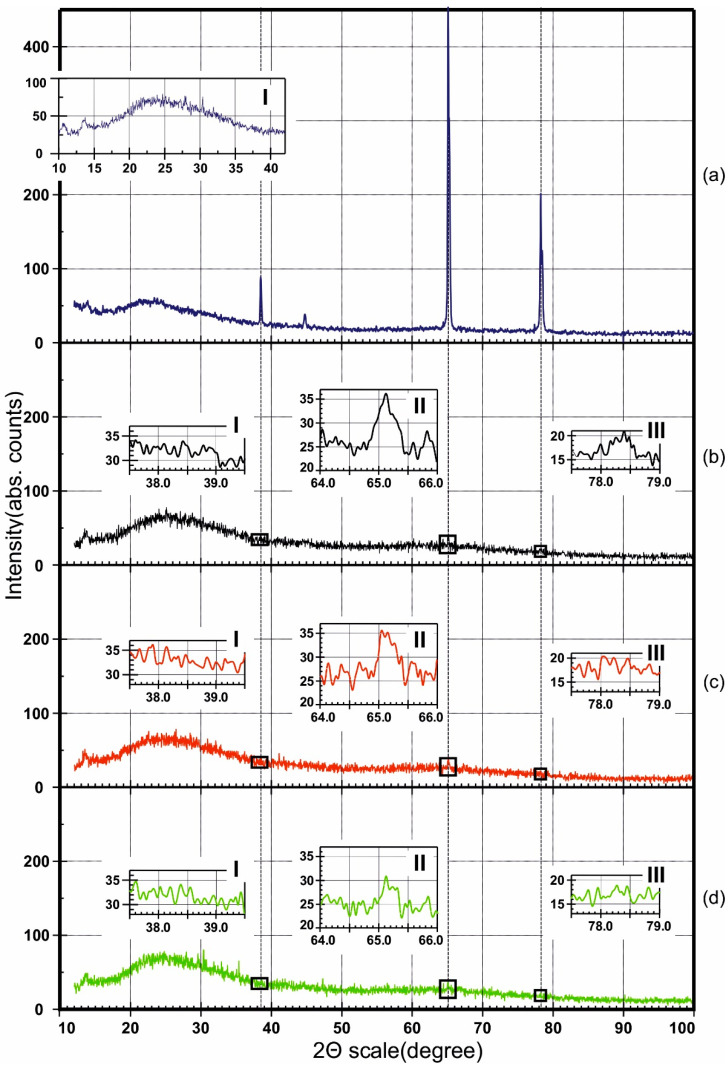
XRD patterns of (**a**) initial aluminum and porous alumina films obtained in (**b**) 0.4, (**c**) 1.0, and (**d**) 2.0 M sulfuric acid solutions, at 4.25, 4.05, and 6.10 mA∙cm^−2^ current densities, respectively.

Interestingly, in Figure 4b, the anodizing reaction exhibits a complex dependence of the current efficiency on the anodic current density calculated from the anodizing time. For the two SA concentrations of 0.4 and 1.0 M, there is first a tendency to the increase of *η* with *j_a_* up to a maximum value, after which *η* drops again; on the contrary, in a 2.0 M solution, *η* slowly rises for all considered *j_a_*. It should be observed that, at the maximum occurring for the former two concentrations, the amount of charge consumed for complete aluminum oxidation is above 100%. This apparently inconsistent result can be justified by assuming that, in reality, complete oxidation of aluminum does not occur, but there is always a certain amount of unreacted metal contained in the oxide volume. This leads to a decrease in the anodizing time in comparison with the theoretically expected value, and—consequently—to an overestimation of *η*. This hypothesis is confirmed by the graphical dependence of the residual amount of aluminum *P_Al_* (Figure 4c) and the corresponding calculations performed on the basis of the XRD results on the PAFs (Figure 7), which result in maximum points for 0.4 and 1.0 M and in the inflection point for 2.0 M, respectively. This result is confirmed by the XRD data for a number of other PAF samples, obtained in SA solutions under different conditions. XRD results for PAF obtained at 1.5, 2.0, 4.05, 4.25, 4.5, 6.1, 5.0, 8.0, and 8.7 mA·cm^−2^ current densities and 0.4, 1.0, and 2.0 M concentrations are shown in Figures S1–S27 of the Supplementary Materials.

In Figure 7a, the XRD of the initial aluminum glued to glass is shown, along with that of a fragment of the glass substrate in the inset. The XRD of the initial aluminum foil contains four peaks with maxima corresponding to 38.51, 44.79, 65.11, and 78.27°. These results are in good agreement with the International Centre for Diffraction Data [69]. In Figure 7b–d, the XRD diffraction patterns of some PAF samples are shown instead.

To identify residual aluminum in the PAFs, three peaks with maxima at 38.51, 65.11, and 78.27° were selected. Figure 7b–d show insets I-III for close-ups of the XRD regions in the 2Θ ranges of 37.5°–39.5°, 64.0°–66.0°, and 77.5°–79.0°, respectively (and more complete generalized results are presented in Figures S1–S27). There appears a tendency to a decrease in the amount of residual aluminum in PAFs obtained in more concentrated SA solutions. For a quantitative assessment, the area of the most intense peak of aluminum at 65.11° was considered, and the % proportion of unreacted (residual) aluminum *P_Al_* was calculated using the formula:

(8) $$P_{Al}=\frac{S_{Al(PAF)}}{S_{Al(Me)}}\cdot 100\%$$

where *S*_*Al*(*PAF*)_ and *S*_*Al*(*Me*)_ are the areas under the 65.11° aluminum peak for the PAFs and the original foil, respectively. The results are plot in Figure 4c. The associated uncertainty was estimated to be ~±4%.

Figure 4c shows the trends in amount of un-oxidized aluminum on the anodizing conditions, a picture that can be combined with the data on anodizing efficiency in Figure 4b, for increased understanding. From Figure 4c, one can observe the occurrence of a maximum in un-oxidized aluminum for samples obtained in 0.4 and 1.0 M SA, and a monotonic increase with *j_a_* for PAFs anodized in 2.0 M SA. Thus, incomplete aluminum oxidation can lead to the fact that the experimental value of charge consumed for the sample anodizing, calculated from the duration of the complete anodizing process, turns out to be less than its theoretical value, or—alternatively—the current efficiency appears higher than 100%. The error in determining the anodizing efficiency can be estimated around 5%, which is explained from the following considerations. Current efficiencies below 100% indicate anodic parallel reactions, i.e., oxygen evolution or/and electrolyte anion oxidation. This means electronic conductivity of the barrier layer. Efficiencies above 100% could mean that cathodic parallel reactions take place, which can be excluded under the given conditions. The highest measured current efficiencies of about 105% can therefore be used to estimate the error of the method [44].

In addition, it should be noted that the presence of un-oxidized metal in depth inside the PAFs obviously affects the electrophysical and optical characteristics of the oxide, and turns out into spectral–luminescent properties of light-emitting materials obtained on its basis using the sol–gel technology [70]. The presence of metallic aluminum in the PAF volume is confirmed by the studies of other authors [32,71,72].

## 4. Conclusions

The paper investigated the dependence of volumetric growth factor, anodizing current efficiency, and amount of residual un-oxidized metal during porous alumina films fabrication by low temperature galvanostatic anodizing of aluminum foil (99.99%) with an initial thickness of 10.5 µm, in aqueous sulfuric acid solutions with 0.4, 1.0, and 2.0 M concentration, in the current density range of 0.5–10 mA·cm^−2^. As a result of the work, the following points emerged:

1. Reduced temperature 280 K electrochemical anodizing of aluminum at extremely low current density and high SA concentration leads to the formation of a non-typical nanostructured porous alumina film consisting of hemispheres with radially diverging pores. The nucleation of hemispheres on the sample surface under such conditions occurs pointwise and, probably, in an orderly manner, and is probably caused by self-organized redistribution of current density over the sample surface.
2. The galvanostatic anodizing rate of aluminum foil in aqueous sulfuric acid solutions increases in proportion to the density of the anodic current.
3. A decrease in sulfuric acid concentration from 2.0 to 0.4 M does not affect the anodizing rate significantly.
4. The volumetric growth factor increases with an increase in current density from 0.5 to 10 mA·cm^−2^, which is caused by an increase in sulfuric acid anions embedded in the porous alumina film, a decrease in the anodizing time and, as a consequence, a decrease in the oxide dissolution rate. The volumetric growth factor decreases with an increase in the sulfuric acid concentration from 0.4 to 2.0 mA·cm^−2^, reaching values from 1.67 to 1.26, which can be explained by an increase in the oxide dissolution rate with an increase in SA concentration.
5. The dependence of porous alumina film volumetric growth factor on the anodic current density is characterized by two saturation regions, the position of which depends on the electrolyte concentration, which are probably related to the sulfuric acid dibasicity and stepwise dissociation.
6. An aluminum phase was identified in the porous alumina film samples. There is a tendency towards a decrease in the content of residual aluminum with an increase in the electrolyte concentration. The amount of un-oxidized metal has a maximum in the current density region of 4–4.5 mA·cm^−2^ for porous alumina films obtained in 0.4 and 1.0 M sulfuric acid and a monotonic increase with increase in the current density for porous alumina films obtained in 2.0 M sulfuric acid.
7. The dependence on the current density for the efficiency of aluminum anodizing in sulfuric acid solutions has a maximum value above 100%, which is explained by the presence of residual metal in the porous alumina films, confirmed by the results of XRD and SEM.

## Figures and Tables

**Table 1:** Best fitting parametric values and standard errors from model Equations (5)–(7) for anodizing time *τ*, anodizing rate *V_a_*, and anodic voltage *E_a_*, respectively, for different sulfuric acid concentrations.

Equation Used	Fitting Results	Sulfuric Acid Concentration (M)		
		0.4	1.0	2.0
5	*a* (s)	−1.99 × 10^5^ ± 9.61 × 10^4^	−1.68 × 10^5^ ± 5.16 × 10^4^	−1.42 × 10^5^ ± 3.06 × 10^4^
	*b* (cm^2^ mA^−1^)	13.2 ± 6.1	11.0 ± 3.2	9.38 ± 1.88
	*R^2^*	0.99423	0.99647	0.99628
6	*A* (μm s^−1^)	3.76 × 10^−5^ ± 1.18 × 10^−4^	−3.36 × 10^−4^ ± 1.93 × 10^−4^	−3.59 × 10^−6^ ± 1.11 × 10^−4^
	*B* (10^11^ μm^3^ C^−1^)	6.08 × 10^−4^ ± 1.04 × 10^−4^	9.63 × 10^−4^ ± 1.72 × 10^−4^	6.24 × 10^−4^ ± 8.20 × 10^−5^
	*C* ( )	1.07 ± 9.18 × 10^−2^	0.83 ± 7.58 × 10^−2^	1.05 ± 5.37 × 10^−2^
	*R^2^*	1	1	1
7	*α* (V)	19.3 ± 1.7	14.4 ± 1.3	7.02 ± 1.36
	*β* (V)	4.49 ± 1.07	4.47 ± 0.70	5.77 ± 0.60
	*γ* (mA·cm^−2^)	−0.547 ± 0.292	−0.358 ± 0.266	0.085 ± 0.237
	*R* ^2^	0.99918	0.99906	0.99876

## Data Availability

Excluded.

## Supplementary Materials

The following are available online at https://www.mdpi.com/1996-1944/14/4/767/s1: Figures S1–S27: X-ray diffraction analysis results of the porous alumina films obtained at 1.5, 2.0, 4.05, 4.25, 4.5, 6.1, 5.0, 8.0, 8.7 mA·cm^−2^ current densities and 0.4, 1.0, 2.0 M SA solution concentrations.
