# Supplementary material for: Porous Alumina Films Fabricated by Reduced Temperature Sulfuric Acid Anodizing: Morphology, Composition and Volumetric Growth
Source: Materials (Basel). 2021 Feb 6;14(4):767. doi: 10.3390/ma14040767 (PMC7915223; doi:10.3390/ma14040767)
Supplement: Supplementary file 1 [file materials-14-00767-s001.pdf]

materials

Supplementary Materials

Porous Alumina Films Fabricated by Reduced Temperature Sulfuric Acid Anodizing: Morphology, Composition and Volumetric Growth

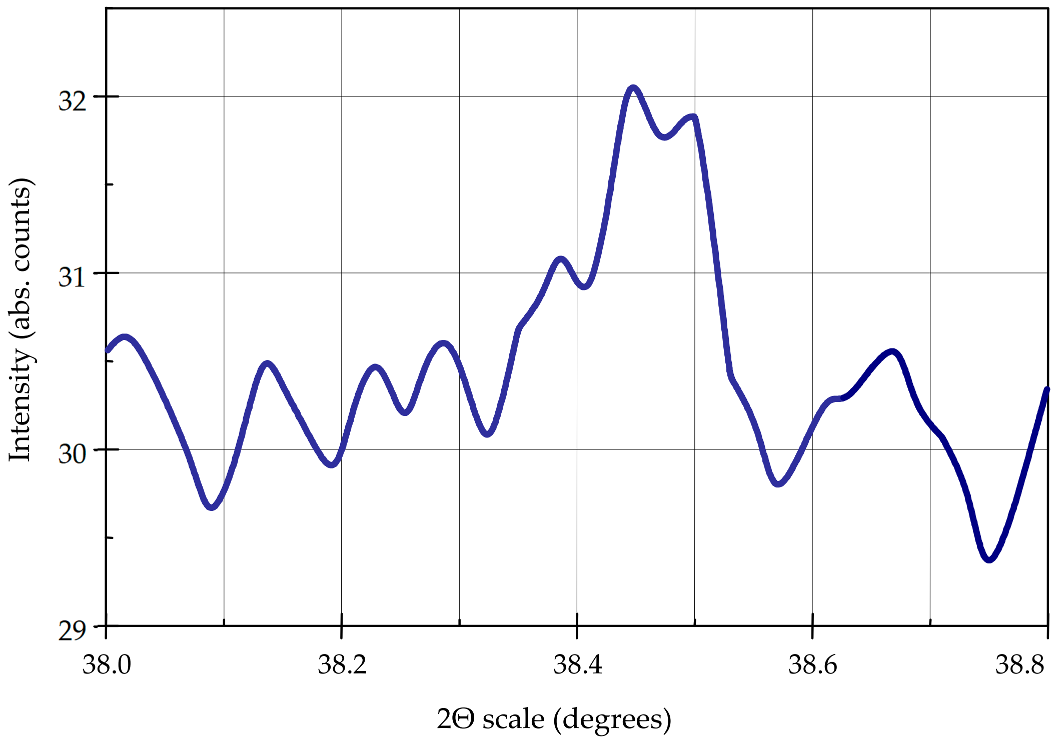

Figure S1. X-ray diffraction analysis of porous alumina film obtained in 0.4 M sulfuric acid solution at 1.5 mA·cm^{-2} current density.

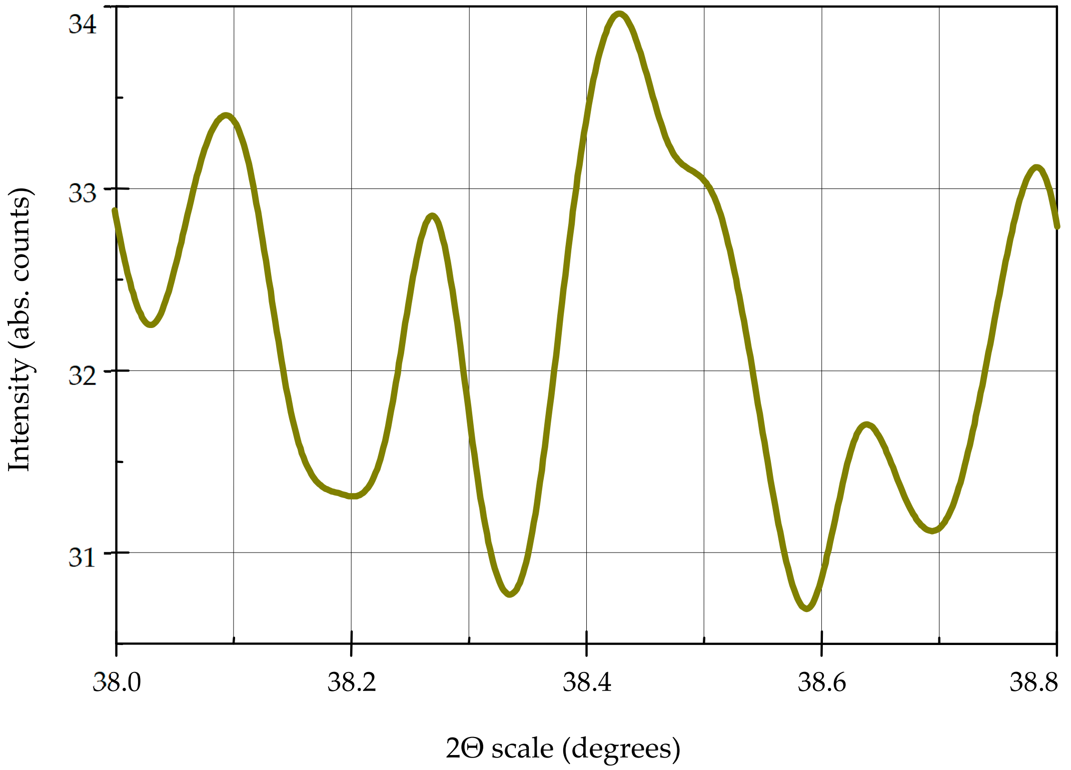

Figure S2. X-ray diffraction analysis of porous alumina film obtained in 0.4 M sulfuric acid solution at 4.25 mA·cm^{-2} current density.

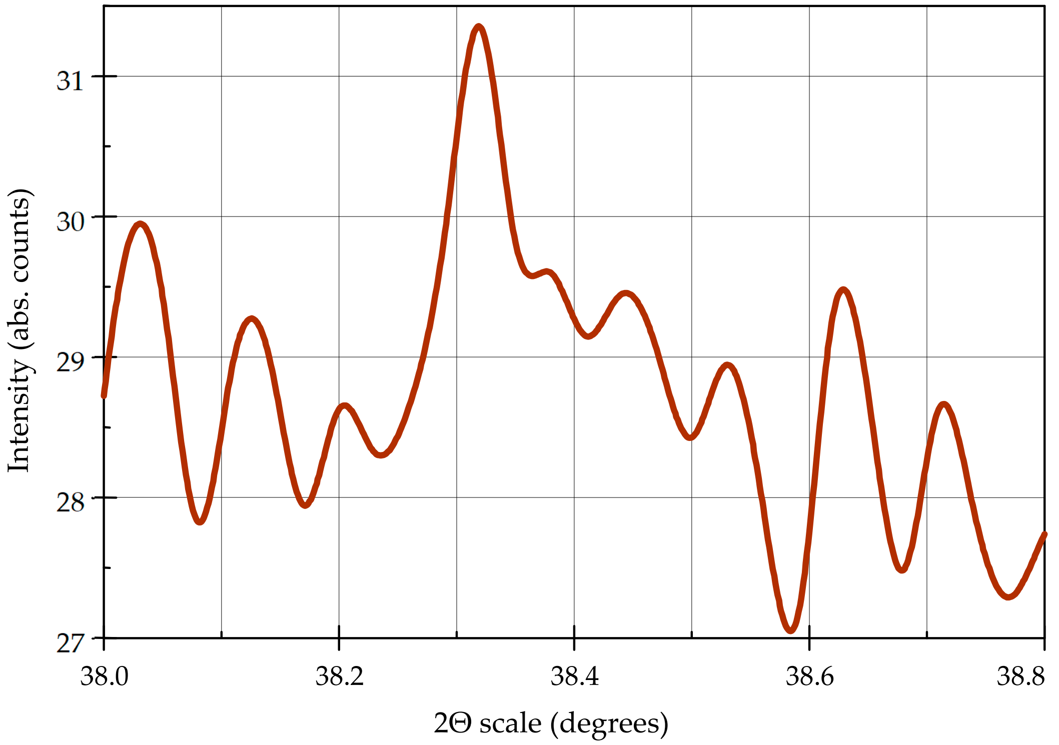

Figure S3. X-ray diffraction analysis of porous alumina film obtained in 0.4 M sulfuric acid solution at 5.0 mA·cm^{-2} current density.

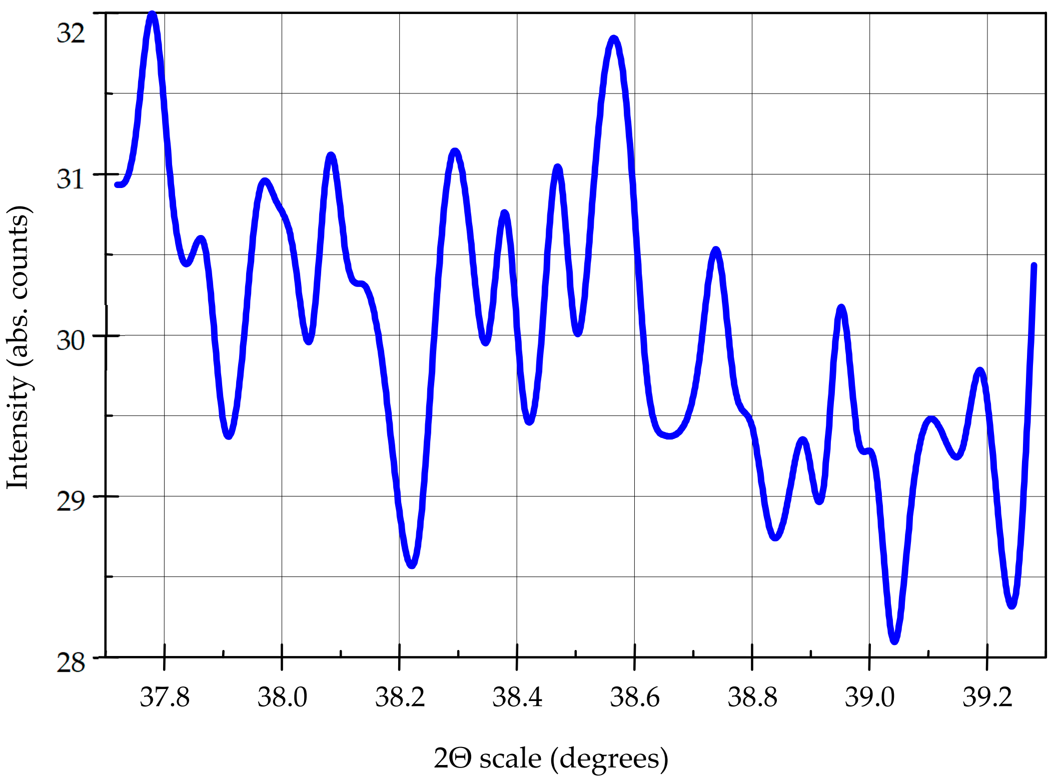

Figure S4. X-ray diffraction analysis of porous alumina film obtained in 1.0 M sulfuric acid solution at 2.0 mA·cm^{-2} current density.

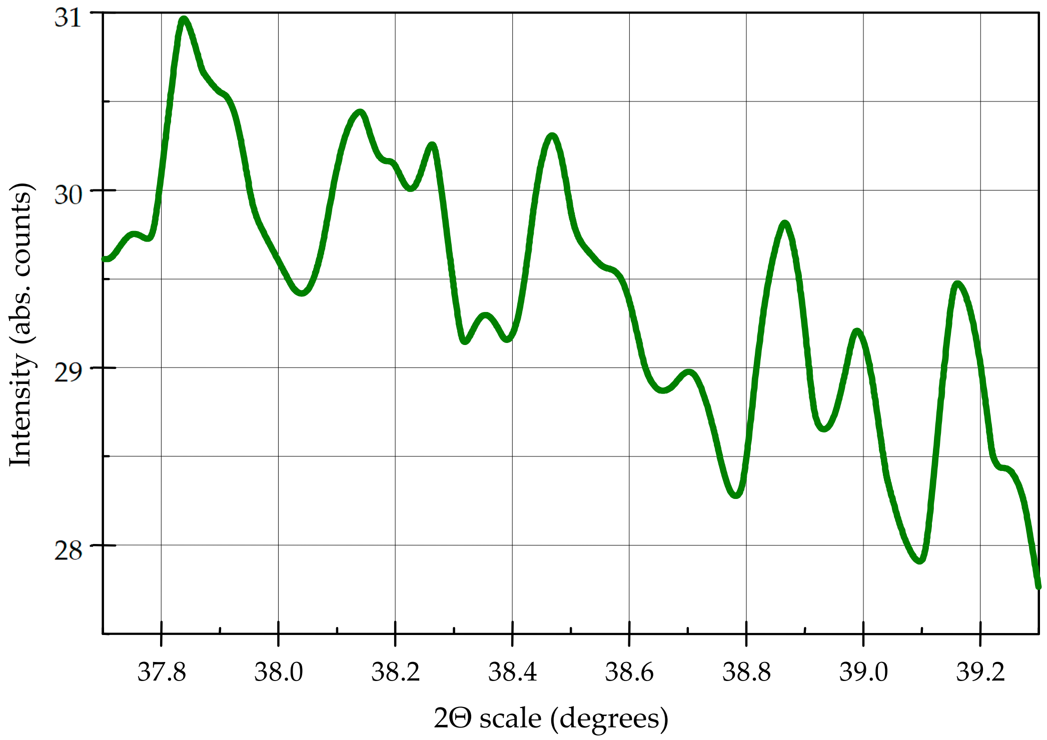

Figure S5. X-ray diffraction analysis of porous alumina film obtained in 1.0 M sulfuric acid solution at 4.05 mA·cm^{-2} current density.

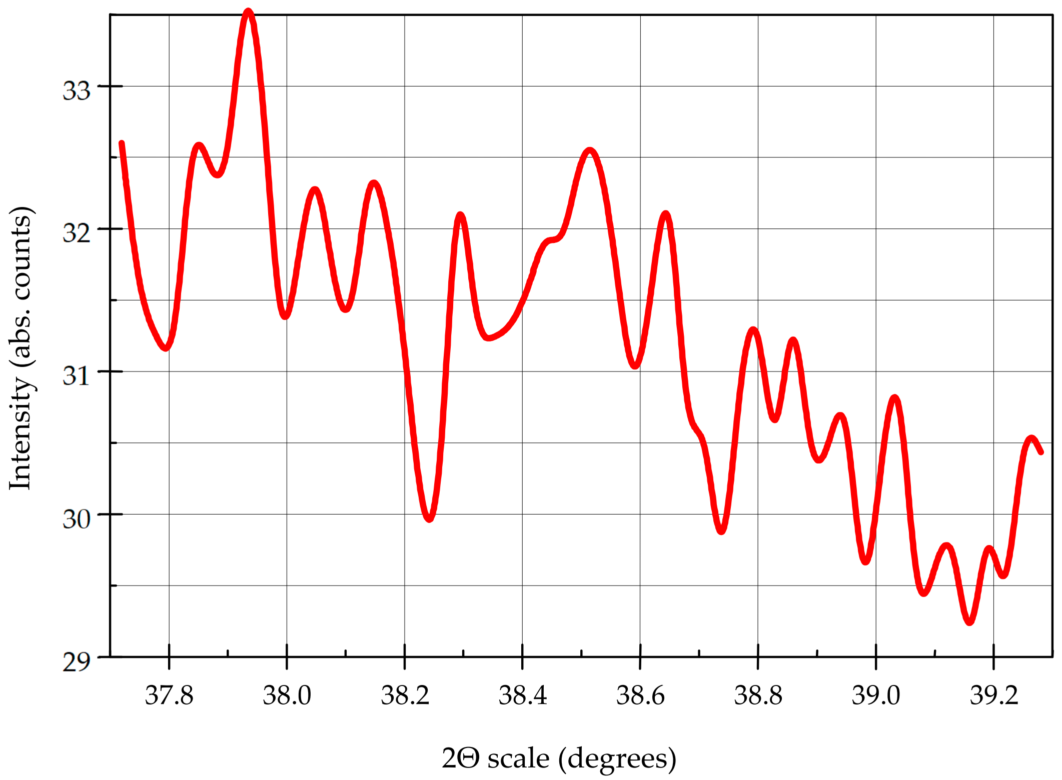

Figure S6. X-ray diffraction analysis of porous alumina film obtained in 1.0 M sulfuric acid solution at 8.0 mA·cm^{-2} current density.

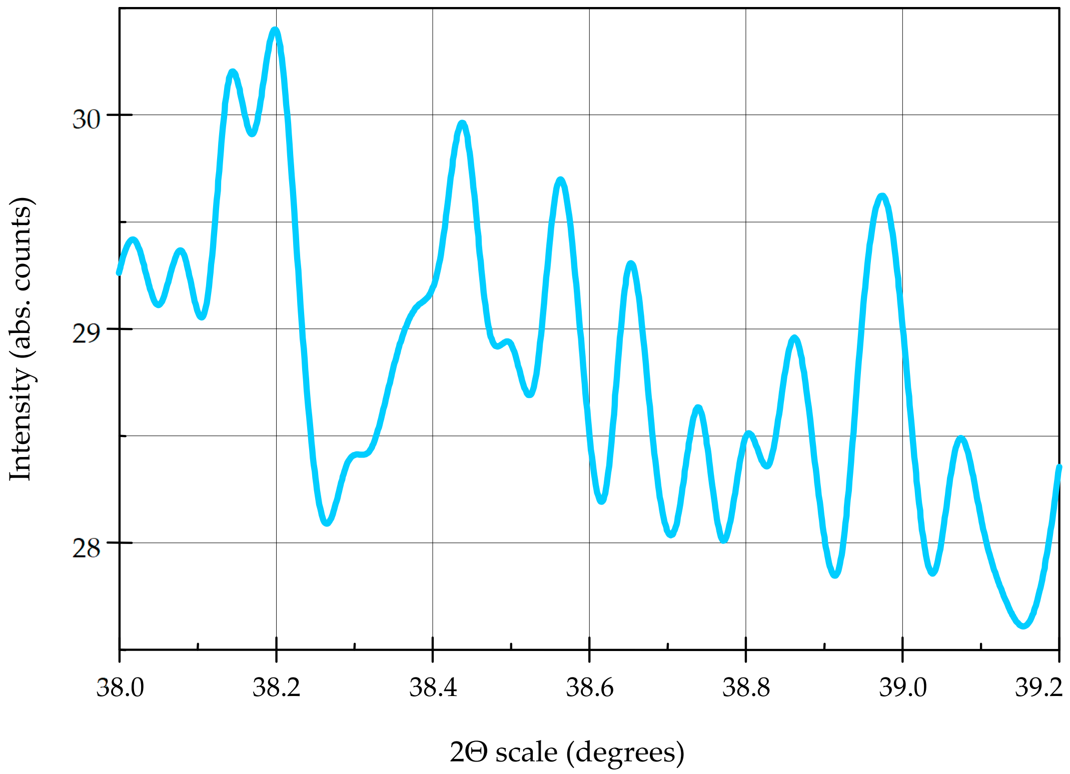

Figure S7. X-ray diffraction analysis of porous alumina film obtained in 2.0 M sulfuric acid solution at 1.5 mA·cm^{-2} current density.

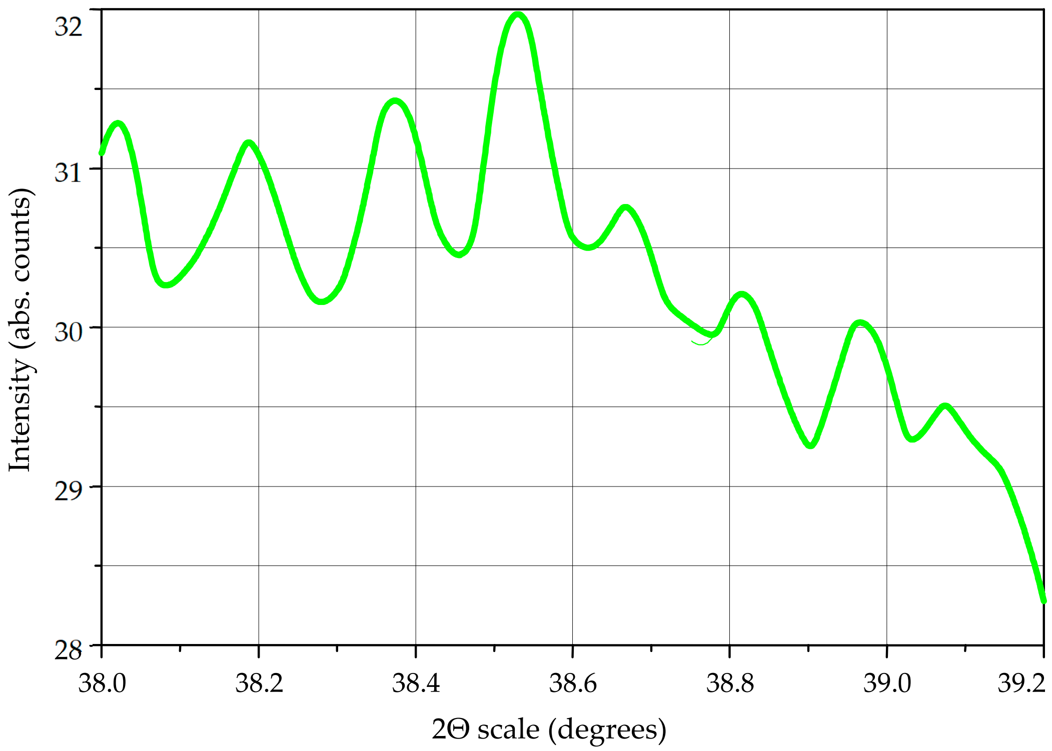

Figure S8. X-ray diffraction analysis of porous alumina film obtained in 2.0 M sulfuric acid solution at 6.1 mA·cm^{-2} current density.

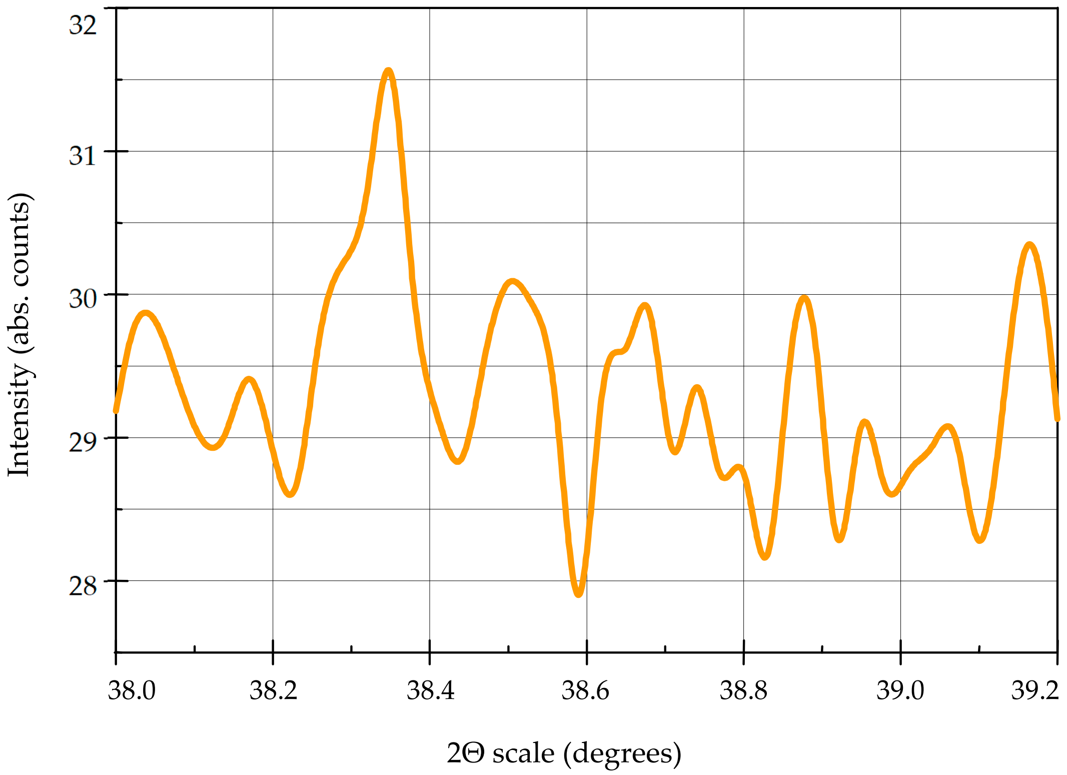

Figure S9. X-ray diffraction analysis of porous alumina film obtained in 2.0 M sulfuric acid solution at 8.7 mA·cm^{-2} current density.

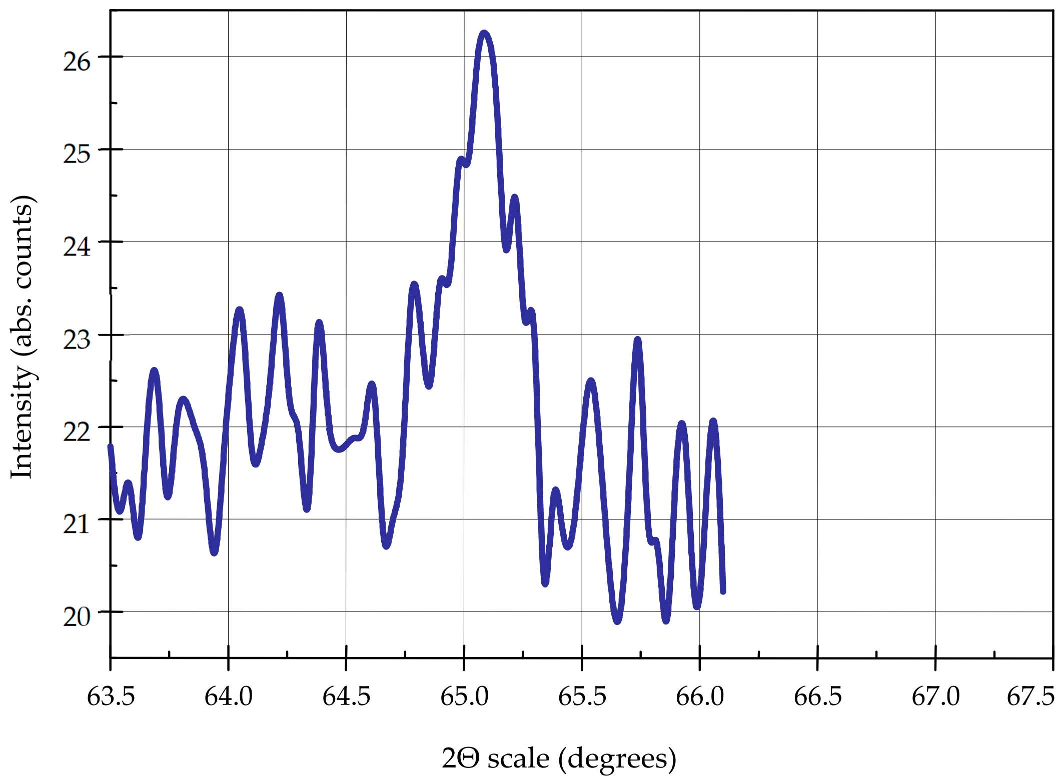

Figure S10. X-ray diffraction analysis of porous alumina film obtained in 0.4 M sulfuric acid solution at 1.5 mA·cm^{-2} current density.

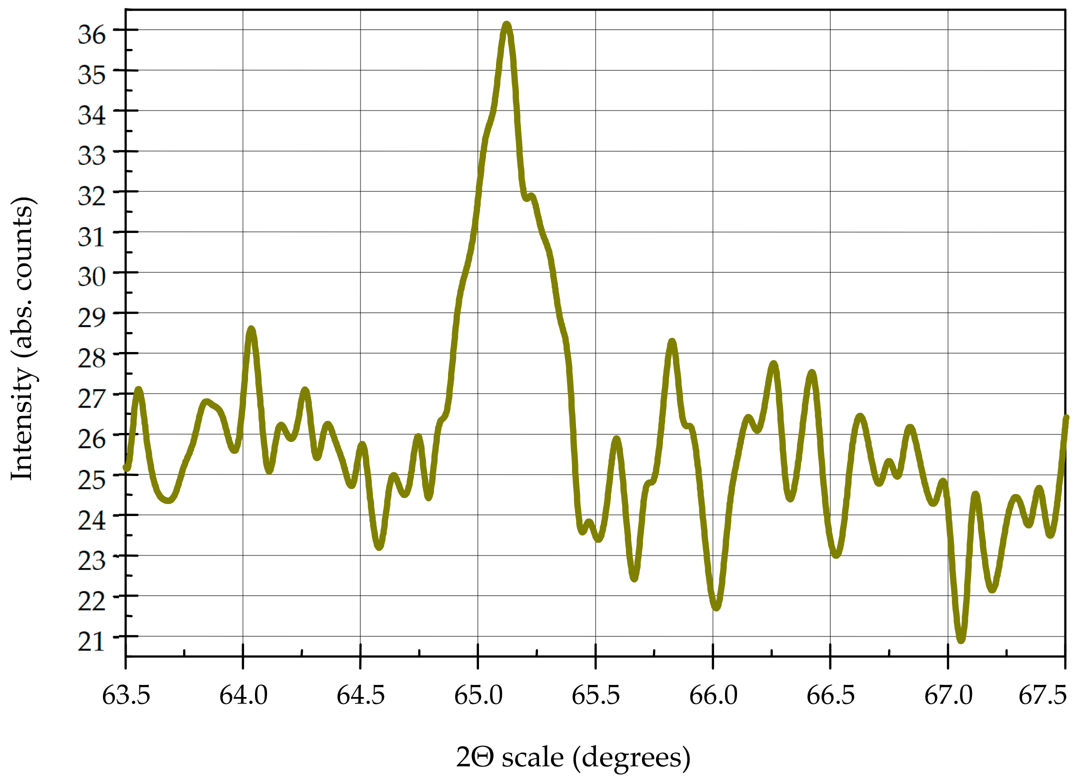

Figure S11. X-ray diffraction analysis of porous alumina film obtained in 0.4 M sulfuric acid solution at 4.25 mA·cm^{-2} current density.

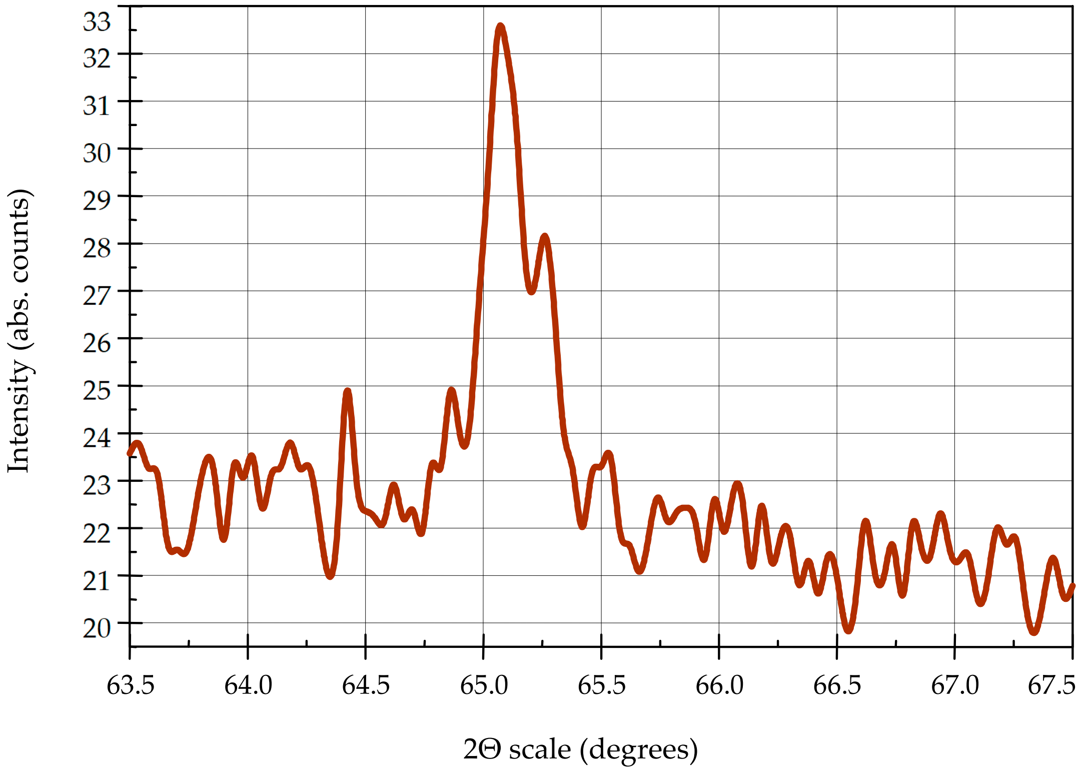

Figure S12. X-ray diffraction analysis of porous alumina film obtained in 0.4 M sulfuric acid solution at 5.0 mA·cm^{-2} current density.

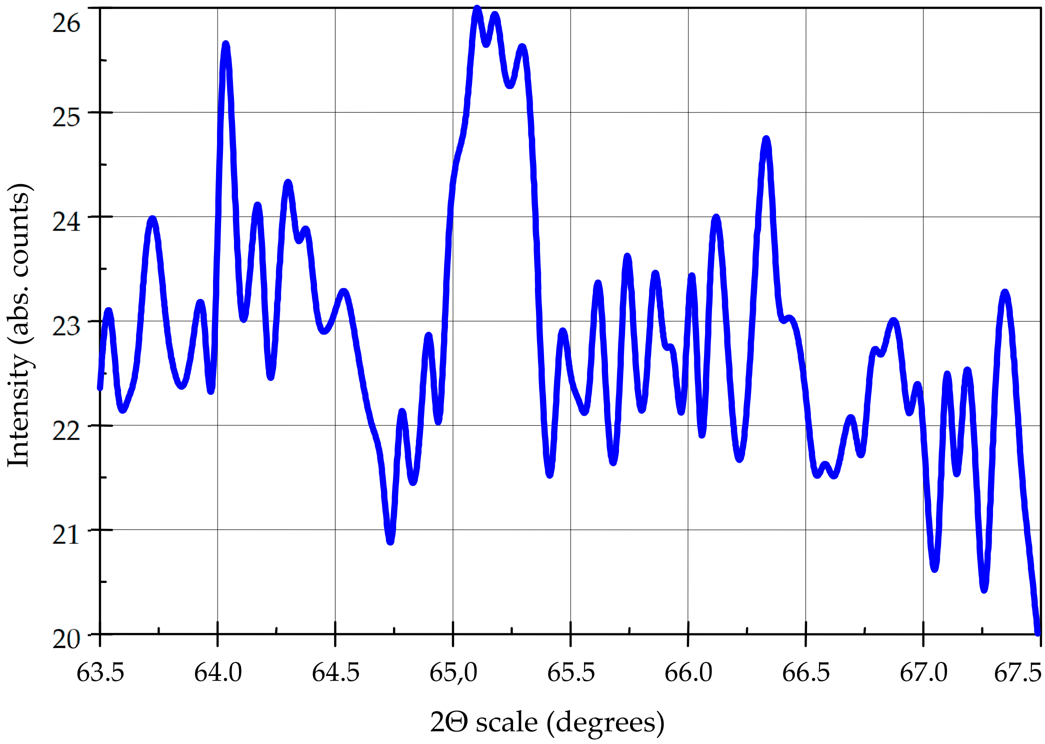

Figure S13. X-ray diffraction analysis of porous alumina film obtained in 1.0 M sulfuric acid solution at 2.0 mA·cm^{-2} current density.

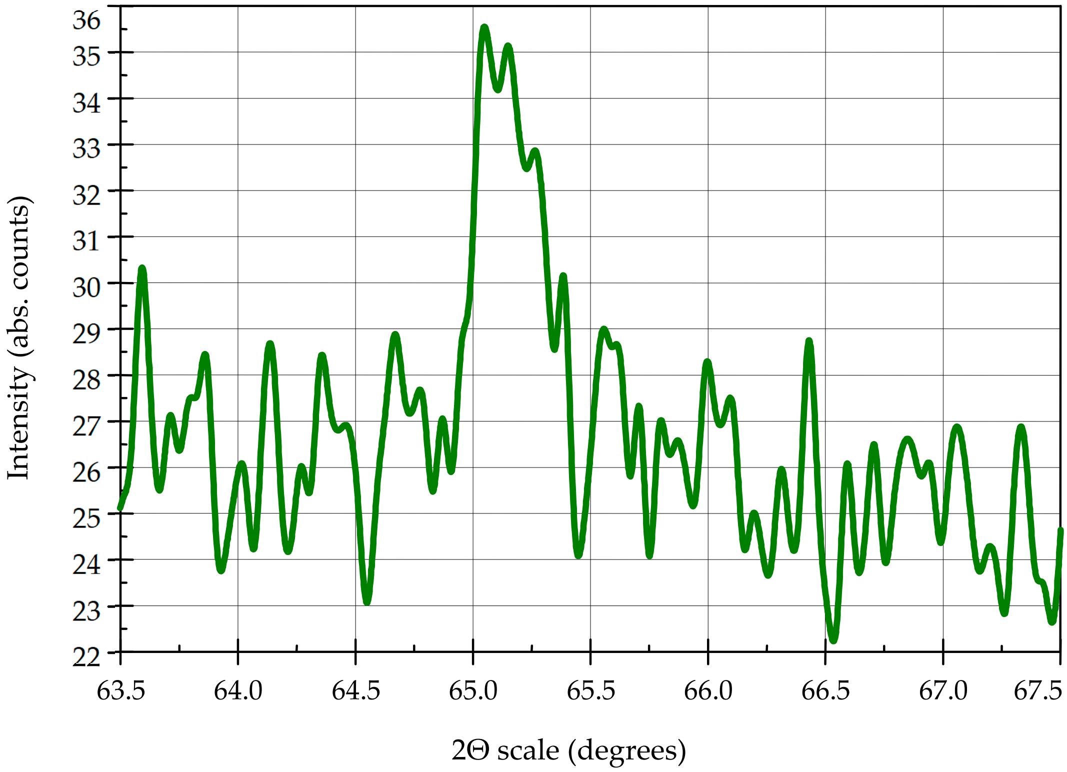

Figure S14. X-ray diffraction analysis of porous alumina film obtained in 1.0 M sulfuric acid solution at 4.05 mA·cm^{-2} current density.

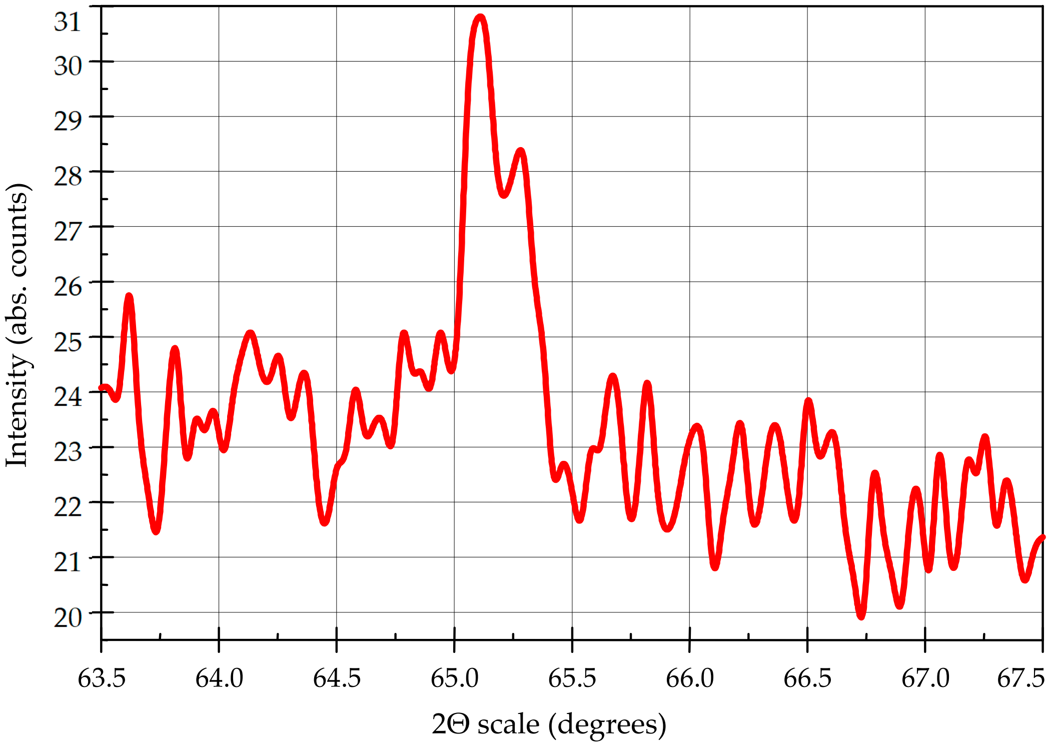

Figure S15. X-ray diffraction analysis of porous alumina film obtained in 1.0 M sulfuric acid solution at 8.0 mA·cm^{-2} current density.

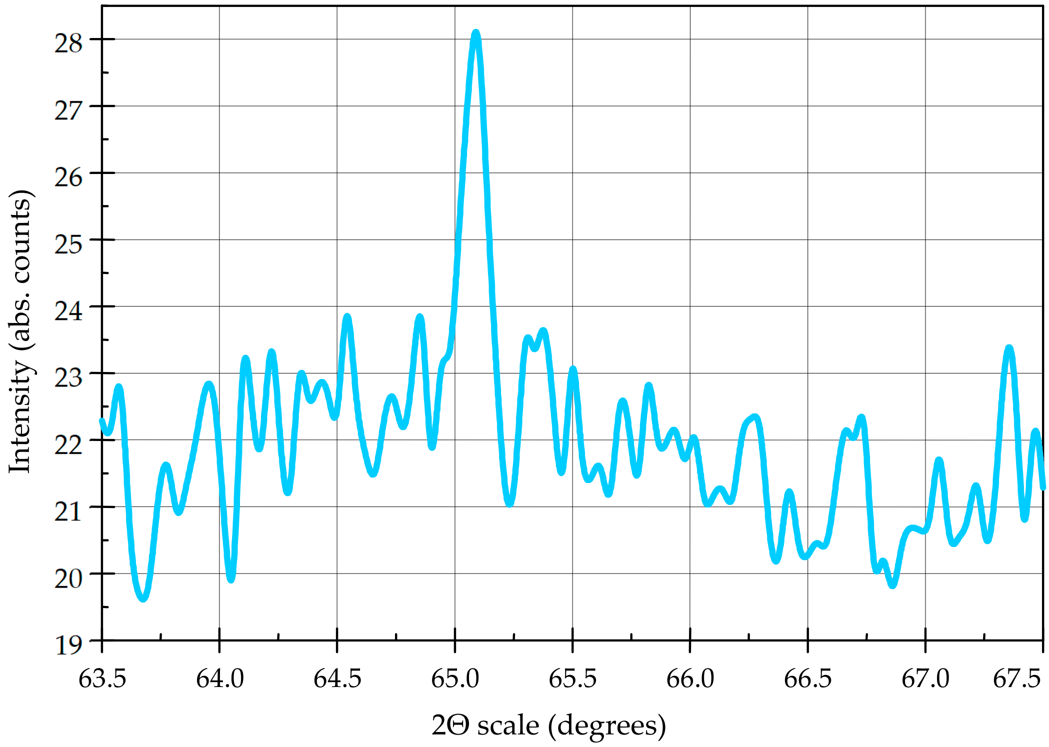

Figure S16. X-ray diffraction analysis of porous alumina film obtained in 2.0 M sulfuric acid solution at 1.5 mA·cm^{-2} current density.

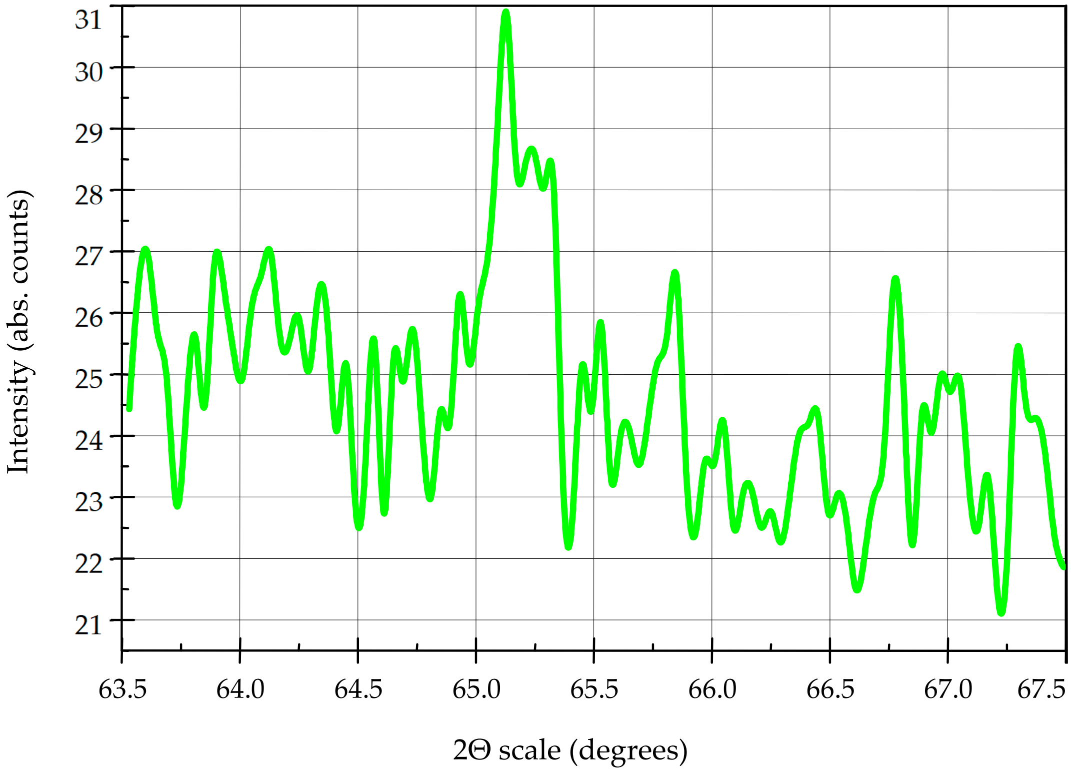

Figure S17. X-ray diffraction analysis of porous alumina film obtained in 2.0 M sulfuric acid solution at 6.1 mA·cm^{-2} current density.

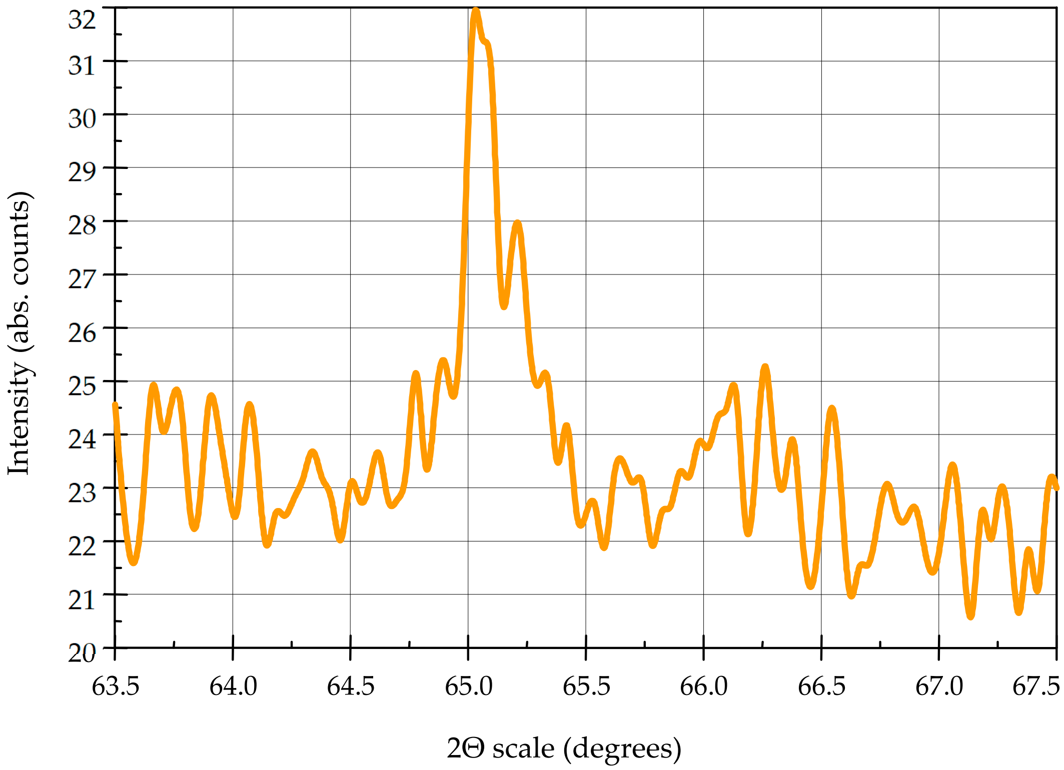

Figure S18. X-ray diffraction analysis of porous alumina film obtained in 2.0 M sulfuric acid solution at 8.7 mA·cm^{-2} current density.

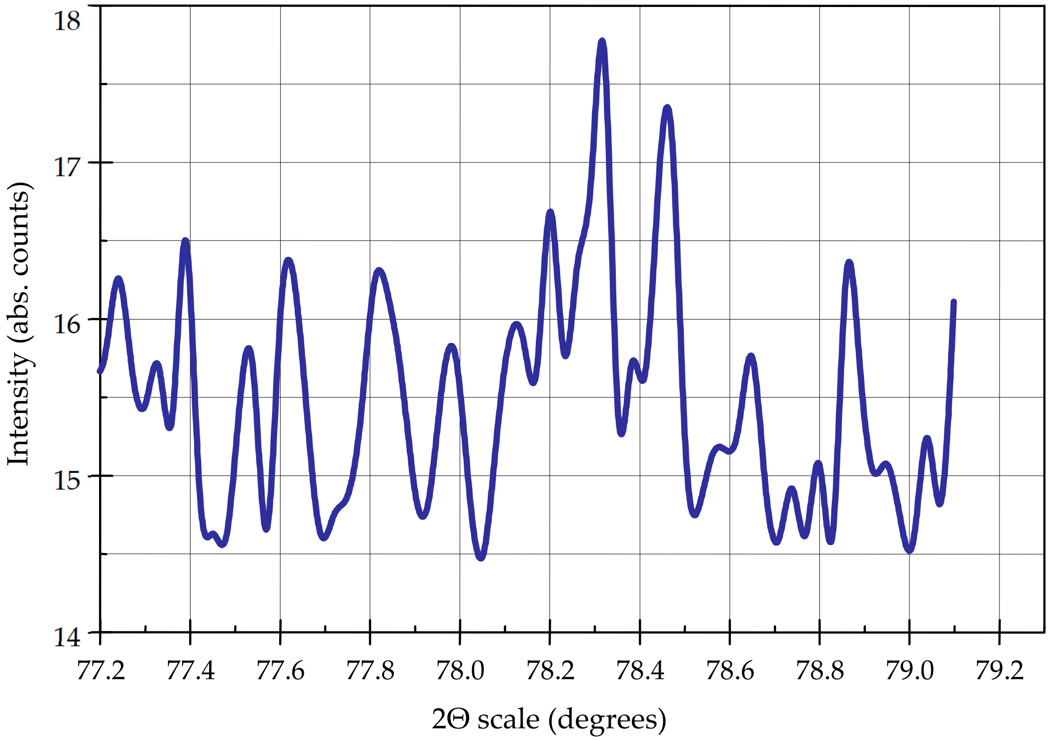

Figure S19. X-ray diffraction analysis of porous alumina film obtained in 0.4 M sulfuric acid solution at 1.5 mA·cm^{-2} current density.

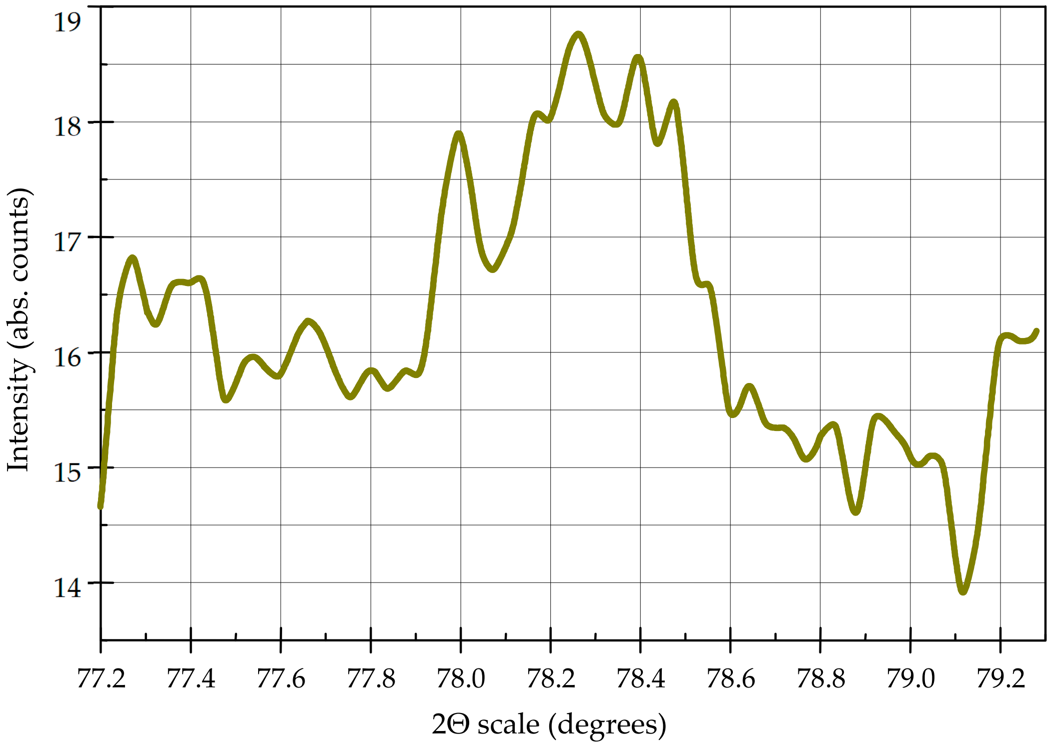

Figure S20. X-ray diffraction analysis of porous alumina film obtained in 0.4 M sulfuric acid solution at 4.25 mA·cm^{-2} current density.

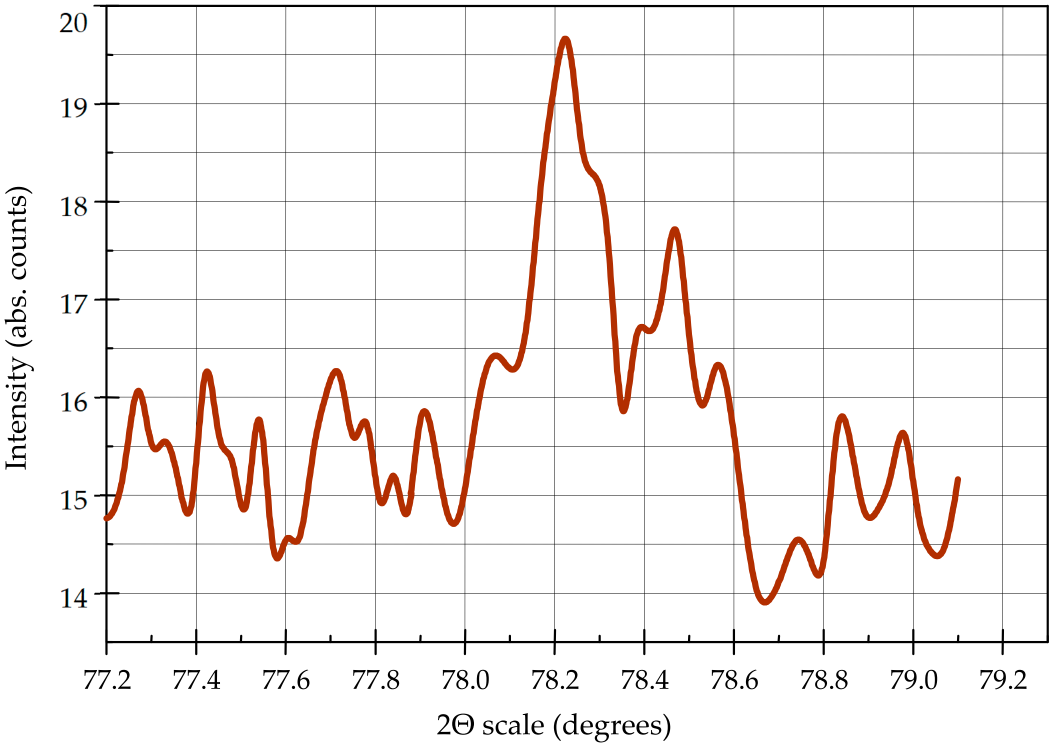

Figure S21. X-ray diffraction analysis of porous alumina film obtained in 0.4 M sulfuric acid solution at 5.0 mA·cm^{-2} current density.

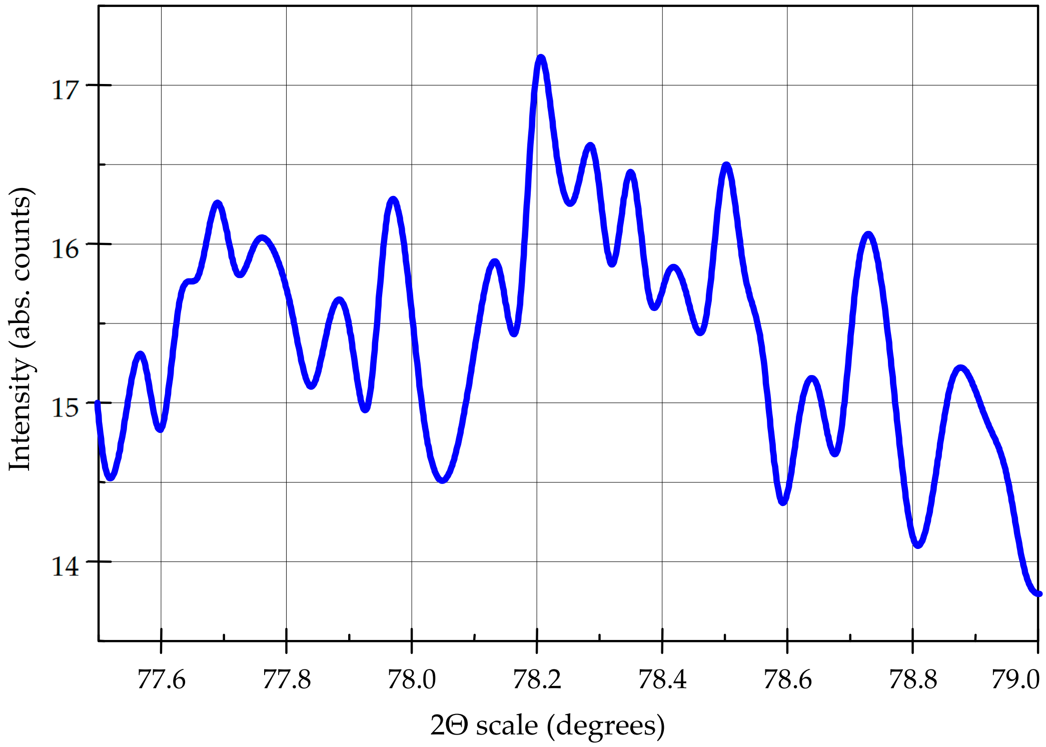

Figure S22. X-ray diffraction analysis of porous alumina film obtained in 1.0 M sulfuric acid solution at 2.0 mA·cm^{-2} current density.

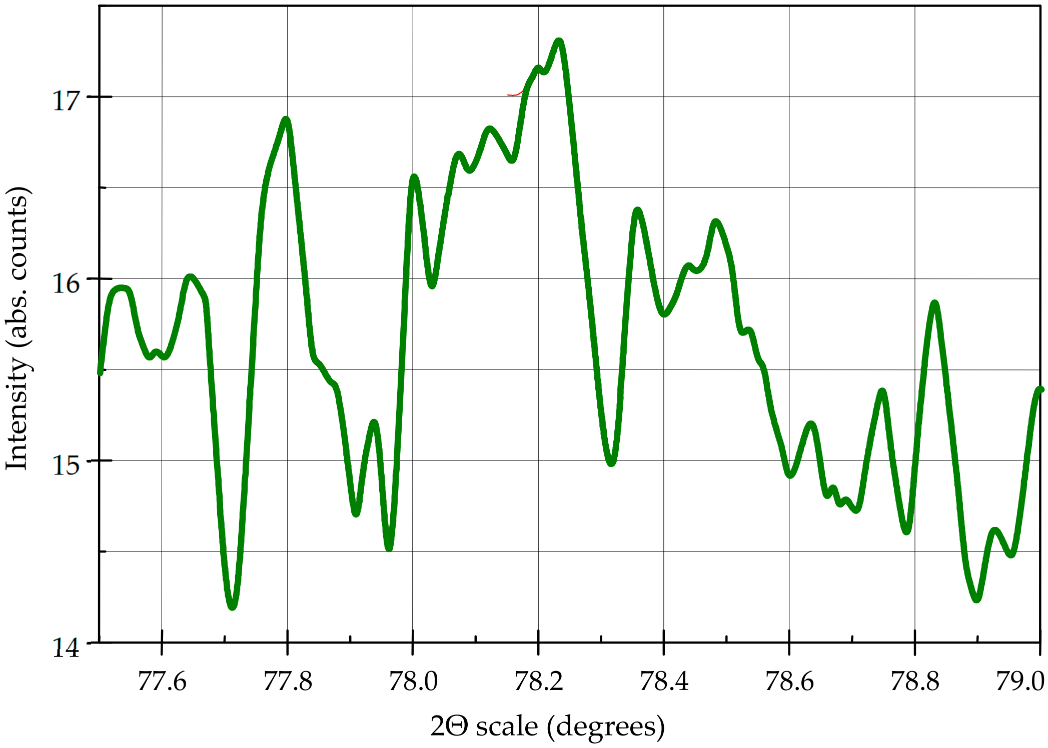

Figure S23. X-ray diffraction analysis of porous alumina film obtained in 1.0 M sulfuric acid solution at 4.05 mA·cm^{-2} current density.

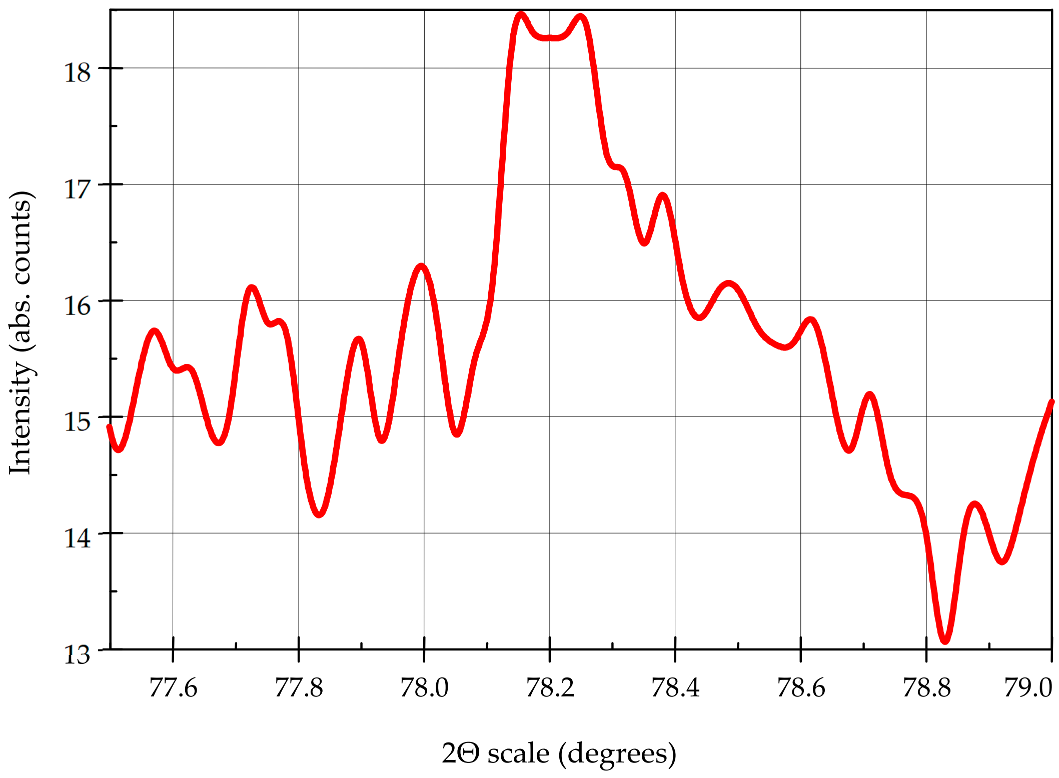

Figure S24. X-ray diffraction analysis of porous alumina film obtained in 1.0 M sulfuric acid solution at 8.0 mA·cm^{-2} current density.

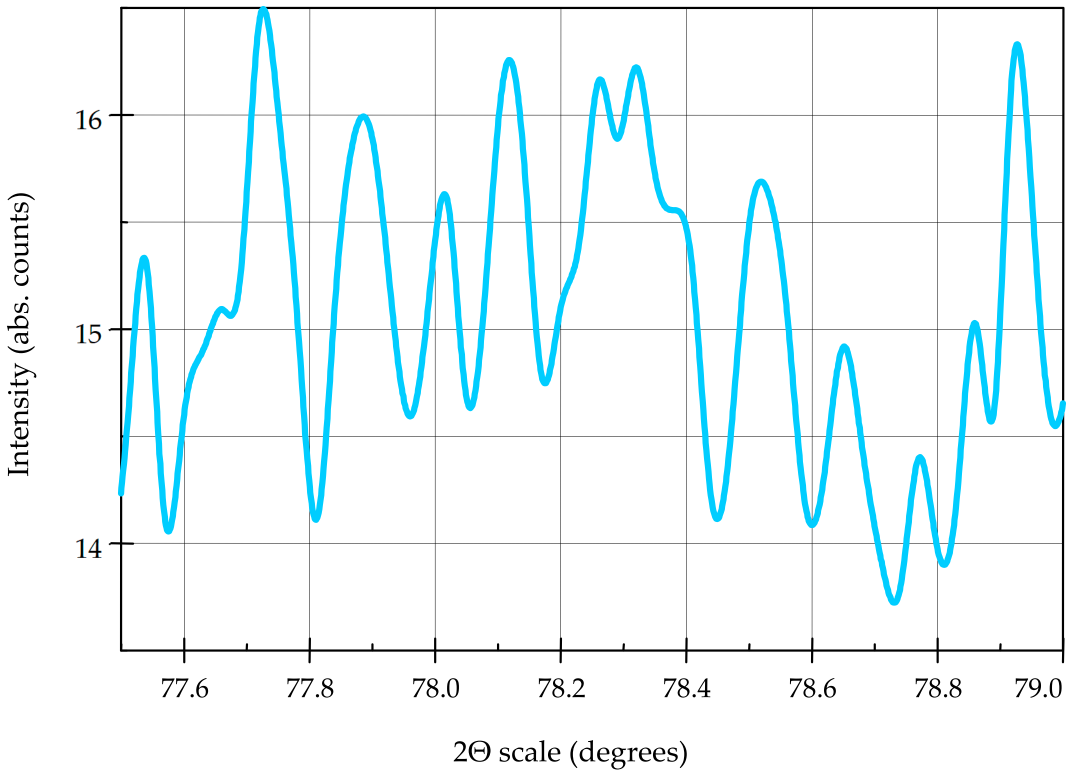

Figure S25. X-ray diffraction analysis of porous alumina film obtained in 2.0 M sulfuric acid solution at 1.5 mA·cm^{-2} current density.

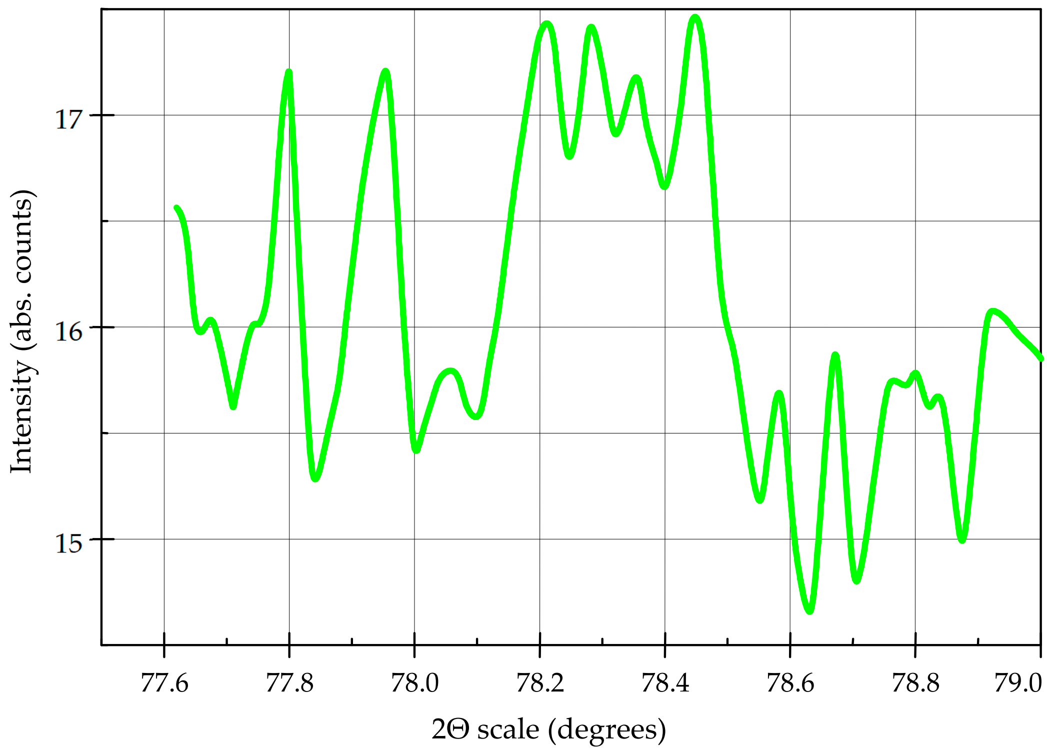

Figure S26. X-ray diffraction analysis of porous alumina film obtained in 2.0 M sulfuric acid solution at 6.1 mA·cm^{-2} current density.

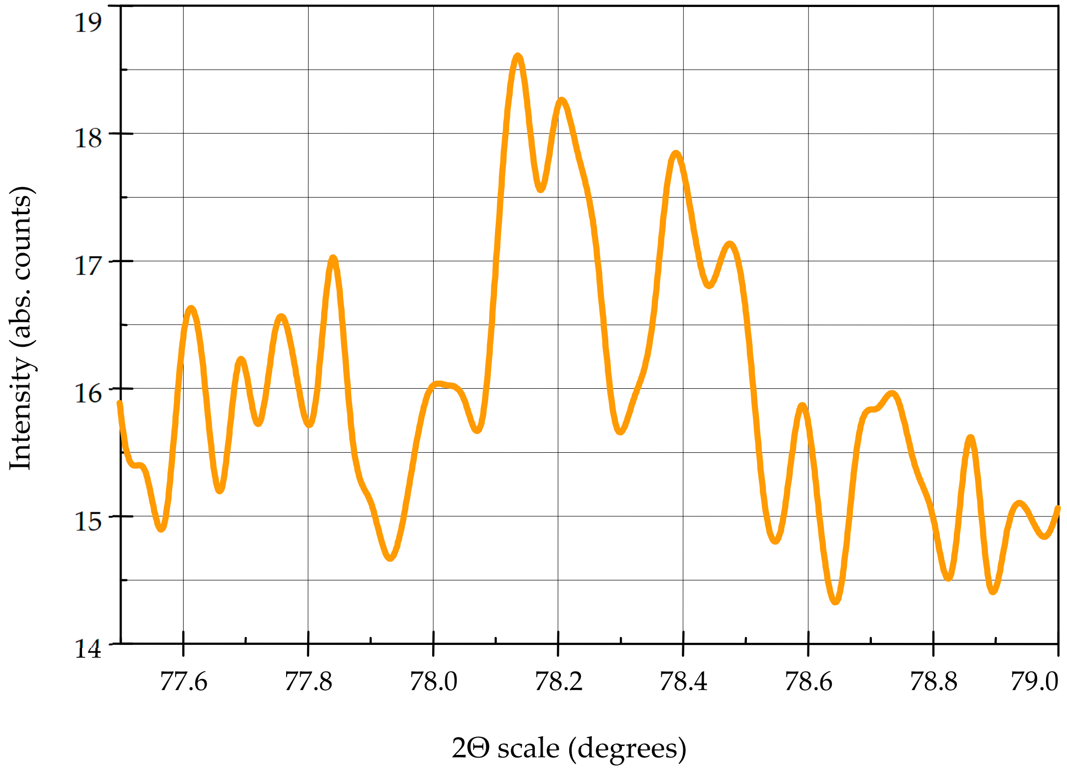

Figure S27. X-ray diffraction analysis of porous alumina film obtained in 2.0 M sulfuric acid solution at 8.7 mA·cm^{-2} current density.
